# Spinal Cord Injury Veterans’ Disability Benefits, Outcomes, and Health Care Utilization Patterns: Protocol for a Qualitative Study

**DOI:** 10.2196/14039

**Published:** 2019-10-04

**Authors:** Denise C Fyffe, Joyce Williams, Paul Tobin, Carol Gibson-Gill

**Affiliations:** 1 Kessler Foundation Spinal Cord Injury/Outcomes and Assessment Research Center West Orange, NJ United States; 2 Rutgers, New Jersey Medical School Newark, NJ United States; 3 VA New Jersey Health Care System Spinal Cord Injury and Disorders Service (128) East Orange, NJ United States; 4 Quality of Life Advisors Fort Myers, FL United States

**Keywords:** veterans, spinal cord injuries, health care

## Abstract

**Background:**

An estimated 42,000 people currently living with chronic spinal cord injury (SCI) are veterans. SCI was a common combat-related injury in the World Wars and Vietnam era and now affects more than 11% of military personnel injured in Operation Iraqi Freedom and Operation Enduring Freedom. The Veterans Benefits Administration primarily offers financial compensation for disabilities sustained or re-aggravated during military service, called service-connected disability compensation. With the overwhelming cost of living with an SCI, this monthly financial compensation can provide service-connected veterans and their families with access to additional supportive resources (eg, assistive devices and personal aide) and maintain their quality of life (QOL). Little is known about personal, health, functional, and QOL outcomes associated with service-connected and nonservice-connected status for veterans living with an SCI.

**Objective:**

The aim of this study is to compare the ways in which Veterans Affairs’ (VA) service-connected and nonservice-connected status may be associated with health and functional outcomes, choice of health care provider, and overall QOL for veterans living with an SCI and their caregivers.

**Methods:**

This cross-sectional qualitative study will gather data using retrospective chart reviews, semistructured interviews, and focus groups. After obtaining institutional review board (IRB) approval, purposeful sampling techniques will be used to recruit and enroll the following key stakeholders: veterans living with an SCI, family caregivers, and SCI health care providers. Concurrent data collection will take place at 2 sites: Veterans Administration New Jersey Healthcare System and Northern New Jersey Spinal Cord Injury System.

**Results:**

This study was funded in July 2015. IRB approval was obtained by November 2016 at both sites. Enrollment and data collection for phase 1 to phase 4 are complete. A total of 69 veterans, 18 caregivers, and 19 SCI clinicians enrolled in the study. Data analyses for these phases are underway. In phase 5, the follow-up focus group activities are scheduled. The final results are expected by the end of 2019.

**Conclusions:**

The factors that contribute to veterans living with SCI seeking and not seeking VA disability compensation benefits are not well understood in rehabilitation research. Triangulation of these data sources will allow us to compare, contrast, and integrate the results, which can be used to develop clinical guidelines, caregiver training, and patient education programs.

**International Registered Report Identifier (IRRID):**

DERR1-10.2196/14039

## Introduction

### Overview

Spinal cord injury (SCI) is among the most devastating and disabling medical conditions affecting wounded members of the military [1-3]. The Department of Veteran Affairs (VA) is the single largest SCI comprehensive health care provider in the nation [4]. There are approximately 42,000 veterans with SCI eligible to receive care at the VA health care facilities [4]. There is also an unknown number of veterans who sustained an SCI following military service that have never used the VA but are eligible to receive health care from the VA. In addition, a portion of these veterans may be entitled to VA disability compensation and ancillary benefits. Veterans Benefits Administration (VBA) disability compensation benefits are designed to provide financial compensation for disabilities sustained or exacerbated during military service as well as secondary disabilities, which are later causally connected to disabilities that incurred during military service; functional deficits that are incurred or aggravated by military service are adjudicated by the VBA as *service-connected* [5,6]. For example, upon leaving the military, a veteran with a lower back injury applies for, and is adjudicated by VBA as having, a service-connected lower back injury. Later in life, the veteran’s spinal cord develops a syringomyelia at the initial level of injury causing a SCI and further functional deficits, which can be adjudicated by VBA as a service-connected disability as well.

As the cost of living with an SCI can be insurmountable, the monthly financial compensation provided to service-connected veterans living with SCI through the VBA can be used to offset the loss of wages. In addition, veterans who are service connected specifically for their spinal cord condition (ie, loss of use feet or hands) may also qualify for additional grants funded through the VBA. These grants promote functional independence by providing resources for the aid and attendance required to maintain the veteran in the least restrictive setting; for example, a VA automobile allowance or specially adapted housing grant where the goal is to help veterans participate in their home life, employment, and social activities that might otherwise be inaccessible and maintain positive quality of life [7,8-10].

Despite VA’s efforts to reduce the financial burden associated with successful rehabilitation, independent living, and community integration through disability benefits, a portion of veterans living with SCI have nonservice-connected disabilities because their disabilities were not incurred or aggravated by their military service [5,6]. On the basis of our literature review, there are no studies to date that have compared the impact of having additional financial resources provided to service-connected veterans living with SCI with nonservice-connected SCI-veterans who do not have these additional financial resources. This is a notable oversight because the views and experiences of the service-connected and nonservice-connected veterans living with SCI may be an invaluable source of insight to the VBA disability compensation program’s effectiveness beyond the mere provision of additional financial resources. Using a community-based participatory design, the proposed study intends to address this gap using qualitative research methods to compare the impact of service-connected status on veterans’ health status, functional outcomes, QOL, family and household, and choice of rehabilitation or medical facilities (ie, VA center or non-VA).

### Background

The Department of Veterans Affairs (VA) estimates approximately 450 newly injured veterans and active-duty members receive rehabilitation at VA’s SCI centers annually [4]. Results from an analysis of the Joint Theater Trauma Registry found the most common combat-related cause of spinal injuries during the Global War on Terrorism are explosions, which account for more than half of the cases, followed by motor vehicle accidents and gunshot wounds [1,11,12].Reports based on data from the National Spinal Cord Injury Database (NSCID) estimate that the average lifetime costs for a 25-year-old individual with high tetraplegia to be more than 3 million dollars, excluding additional opportunity costs such as lost wages, benefits, and productivity [13]. A disproportionate number of individuals living with SCI (62.7%) reside in households with an annual income of US $25,000 or less, and the NSCID reports that only 11.5% of persons with SCI report being employed 11-year after injury [14]. The evidence that socioeconomic disadvantage is common among persons with SCI suggests that this group is at increased risk for poorer health and functional outcomes, given the pervasive negative relationship between socioeconomic disadvantage and health and functional status [15]. Furthermore, these indicators of disadvantage may be exacerbated by the complexity of military service among veterans living with SCI, such as comorbid traumatic brain injury, pain, and posttraumatic stress disorder (PTSD) [16], suggesting that an examination of the provision of financial resources for veterans living with SCI to support health outcomes, functional independence, and QOL is warranted.

The primary goal of VA SCI and Disorders (SCI/D) services is to restore functioning, reduce secondary complications, and promote the health and sustainability of functional independence to maximize QOL after injury [17]. The VA SCI/D System of Care is referred to as a *hub and spoke* system. The VA SCI/D System of Care includes 25 regional SCI/D Centers (known as *hubs*) that provide comprehensive range of care including, inpatient and outpatient rehabilitation, specialty care, and coordinated lifelong continuum of care delivered by interdisciplinary teams [17]. After rehabilitation, most veterans living with SCI return to live in the community [17]. Thus, independent living, community reintegration (eg, functional independence, social participation, and employment access), and QOL are top priorities for VA SCI center rehabilitation [14,17].

The SCI Model Systems (SCIMS) are specialized programs of care in SCI that gather information and conduct research with the goal of improving long-term functional, vocational, cognitive, and QOL outcomes for individuals with SCI. SCIMS, sponsored by the National Institute on Disability, Independent Living, and Rehabilitation Research, Administration for Community Living, US Department of Health and Human Services, supports innovative projects and research in the delivery; demonstration; and evaluation of medical, rehabilitation, vocational, and other services to meet the needs of individuals with SCI. The Northern New Jersey Spinal Cord Injury System (NNJSCIS) was established as a SCIMS in 1990. The NNJSCIS provides a comprehensive continuum of state-of-the-art care for persons with spinal cord injury and their families from the time of injury through rehabilitation and return to the community.

### Veterans Disability Compensation Benefits

VA provides monthly disability compensation benefits to veterans who develop medical conditions and disabilities related to military service; that is, who are deemed *service connected* [5,6,18-19,20]. Veterans seek service connected disability compensation benefits when: (1) they are discharged from the military because of a disability that was incurred or aggravated during military service; (2) a disability manifests itself after the veteran leaves the military but the veteran believes he can prove that its origins occurred in the military (ie, low back pain because the veteran was an infantryman who carried a heavy ruck sack on multiple deployments); (3) a veteran has an earlier service connected disability that results in a worsened disability (eg, service connected knee injury leads to a fall resulting in a SCI); or (4) a veteran is diagnosed with a condition that is presumptively considered service-connected (ie, if a veteran is diagnosed with amyotrophic lateral sclerosis [21] following their military service, it is presumptively considered service-connected and compensable). To qualify for disability compensation benefits, veterans have to submit an disability compensation application and complete a medical assessment to ascertain the functional impact of their disabilities and its impact on a veterans’ *average impairment in earning capacity* [5,6,22-23,18-19]. On the basis of the VBA Schedule for Rating Disabilities, disability ratings range from 0% to 100% in 10% increments (ie, *scheduler ratings*), with a higher percentage of rating equaling a greater functional impairment and amount of disability compensation awarded. Typically, a VA disability rating is derived from an algorithm that combines the individual scheduler ratings of each compensated disability [5,6]. Therefore, two 10% disability ratings do not equal 20%. The algorithm takes into account the number and severity of each scheduler rating and attempts to calculate the overall impact on average earning capacity. Typically, veterans with service-connected SCIs that impact their ability to walk and cause neurogenic bowel and bladder have scheduler ratings of 100% for bilateral loss of use of lower extremities, 60% for neurogenic bladder, and 60% for neurogenic bowel [5,6]. The algorithm combines these ratings to 100% and awards maximum VA disability compensation benefits [5,6]. Unlike Social Security Disability Insurance, which by definition can only be awarded when a disability results in the inability “to engage in any substantial gainful activity by reason of any medically determinable physical or mental impairment(s) which can be expected to result in death or which has lasted or can be expected to last for a continuous period of not less than 12 months,” VA disability compensation benefits are tax-exempt and not automatically discontinued if the veteran returns to work [5,6,22-23,18-19].

### Research Problem

Little is known about the differential impact of service-connected status on the health status, functional outcomes, QOL, and health care utilization patterns of veterans living with SCI. Interestingly, veterans service-connected for PTSD have been found to report high rates of medical impairment, psychiatric symptomatology, and utilization of medical and mental health services [22,23,18]. Furthermore, veterans with PTSD sought service-connected disability compensation of internal factors (eg, tangible needs, need for the problem identification and clarification, and justification and legitimization of *invisible wounds*) and external factors (eg, encouragement from trusted others and professional assistance associated with seeking disability benefits) [19]. These findings suggest there may be a range of factors to consider that may differentially impact veterans living with an SCI based on their service-connected status.

The proposed study intends to address this gap in the literature using qualitative research methods to explore the perspectives of service-connected and nonservice-connected veterans living with SCI, family caregivers as well as SCI clinicians about factors that contribute to these veterans’ health status, functional outcomes, and health care utilization. On the basis of the aforementioned literature, individual/personal factors, socioeconomic, family, and health system factors will be explored. Individual factors such as demographic characteristics (eg, age), cultural beliefs, socioeconomic status (eg, education and income), and health risk behaviors (eg, smoking and alcohol use) have been found to the impact on health status, functional outcomes, and health care utilization in veterans living with SCI [24]. Family caregivers provide assistance that is critical to sustaining health status, functional gains, and access to health care services as Veterans living with SCI return to the community and will provide information about household/community barriers observed across service-connected and nonservice-connected veterans living with SCI [25-27]. SCI health care providers, such as physicians, nurses, social workers, and occupational and physical therapists will provide insights into clinical factors that could contribute to health status, functional outcomes, and health care utilization among veterans living with SCI. Given the high cost of living with an SCI, understanding veterans’ reasons for seeking or not seeking service-connected disability compensation benefits will provide insights about the ways in which veterans’ manage their health, functioning, health care, and QOL.

### Conceptual Model

**Figure 1:**
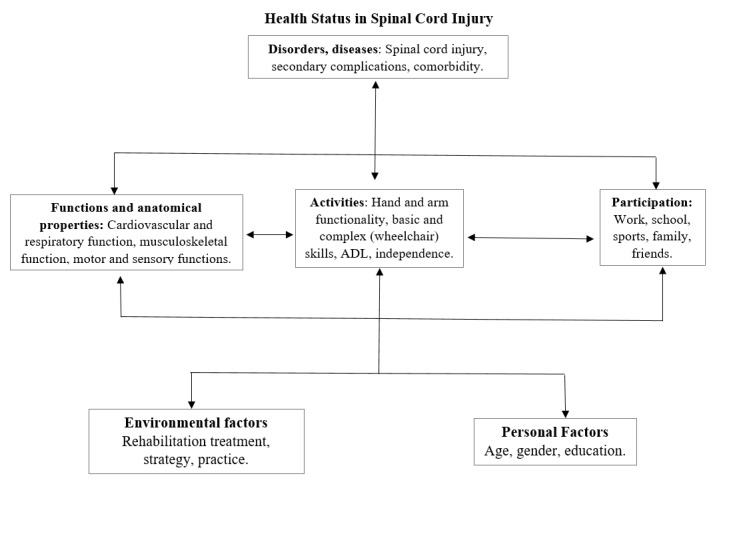
International Classification of Functioning model for spinal cord injury (adapted from de Groot et al, 2009 [27]). ADL: activities of daily living; ICF: International Classification of Functioning, Disability and Health; SCI: spinal cord injury.

This investigation will be guided by the framework of the International Classification of Functioning, Disability, and Health (ICF) [25,26] to examine how service-connection status influences health, function, and health care utilization patterns among veterans living with an SCI. The ICF model conceptualizes disability as an interaction between impairment, functioning, personal factors, and the environment. The ICF can be used to identify, mitigate, or remove societal barriers to full participation of persons with SCI [27-29]. Functioning and disability are viewed as a complex interaction between the impairment of individual, environmental (ie, contextual), and personal factors. Within this framework, SCI is a condition that most often results in impairments such as permanent paralysis. Paralysis then leads to secondary complications, functional limitations, and restrictions to community participation over time. The ICF model serves as a rehabilitation model that will be used to guide the data collection, measuring project outcomes, and designing of clinical guidelines, family interventions, caregiver training, and patient education programs (Figure 1).

### Specific Aims

The proposed project will use qualitative methods to examine the factors associated with outcomes for service-connected and nonservice-connected SCI. Qualitative methods have the advantage of allowing us to address these aims in a manner that is meaningful to individuals who are actively involved in SCI veteran rehabilitation: veterans living with SCI, family caregivers, and SCI clinicians. We propose the following aims:

1. To describe veterans living with SCIs’ reasons for seeking versus not seeking service-connected disability compensation and the factors that influence their choice.
2. To explore the impact of service-connected disability compensation on health status, functional outcomes, QOL, and medical decision making (eg, choice of VA SCI Center versus private sector).
3. To explore the impact of service-connected disability compensation on the family caregivers and households of veterans living with SCI.
4. To explore SCI clinicians’ perspectives on the impact of service-connected disability compensation status on the provision of adequate long-term health care and rehabilitation for veterans living with SCI.
5. To develop a set of practice and policy recommendations about the impact of service-connection status of veterans living with SCI on clinical and policy guidelines, family interventions, caregiver training, and patient education programs.

The proposed work is significant because it will provide new knowledge about veterans living with SCI with and without service connected disability compensation in the realm of family caregiver support, access to community resources, personal factors, and health behaviors, including patient-provider relationships and their impact on health status, functional outcomes, and QOL. The findings will describe areas of care considered priorities for veterans living with SCI and families that must be clearly integrated into clinical care to support the successful maintenance of health status, functional outcomes, and QOL.

## Methods

The study protocol, research team and data collection instruments were reviewed and approved by the institutional review board (IRB) at each study site as well as US Army Medical Research and Materiel Command`s Office of Research Protections, Human Research Protection Office.

### Research Design

This cross-sectional qualitative study will use a community-based participatory approach to examine the reasons for seeking disability compensation benefits, factors associated with outcomes, and choice of health care facility among veterans living with SCI with and without service-connected disability benefits. Community-Based Participatory Research represents a collaborative process between researchers and community partners; builds on the unique strengths, knowledge, and resources within a given community by employing local knowledge in the understanding of health problems and their potential solutions; and facilitates collaboration throughout all phases of the research [30].We will collaborate and gather data from veterans living with SCI, caregivers, and SCI professionals who will serve as *experts* to explore the health status, functional outcomes, and QOL of veterans living with SCI with different types of disability benefits and who decide to receive their health care from VA centers and non-VA.

#### Interdisciplinary Project Team

To achieve these aims, we assembled a highly productive and interdisciplinary team of SCI/D researchers and community advisors with expertise in qualitative research methods, VA SCI *hub and spoke* system of care, private care sector of SCI care, caregiving, and SCI research.

#### Participants

The study will collect data from 3 groups of participants involved in sustaining the health status, functional independence, and QOL of veterans living with SCI who are impacted by the type of disability benefits and health care utilization patterns:

#### Spinal Cord Veterans

An equal number of veterans living with SCI receiving clinical services or who have previously participated in research at the East Orange Campus of the VA New Jersey Health Care System (VANJHCS) or Northern New Jersey SCI Model System (comprised the Kessler Foundation and Kessler Institute for Rehabilitation) will participate in phase 1 (n=15 per site) and 2 (n=30 per site) of the study (described below).

#### Inclusion Criteria

Inclusion criteria are being male, veterans (or served on active duty service in the US Armed Forces), at least 18 years of age, SCI that occurred at least 1 year ago, and receiving health care/rehabilitation at the participating VANJHCS and/or a participating Spinal Cord Injury Model System site or Spinal Cord Injury Model System (SCIMS).

#### Exclusion Criteria

Exclusion criteria are inability to communicate because of neurological impairment (eg, dementia or aphasia); identifying as a female. We limited the scope of the study to patients to male veterans living with SCI because there are not likely to be sufficient numbers of female veterans living with SCI to be able to recruit a meaningful sample of them, given a larger proportion of people with SCI and veterans are males and thus beyond the scope of this project.

#### Spinal Cord Injury Veteran Family Caregivers

SCI often results in physical limitations such that receiving assistance from others is critical to maintaining health and facilitating full societal integration. The help received ranges from assistance with basic daily activities such as bowel and bladder management and dressing, as well as instrumental activities of daily living, including managing household finances, shopping, or transportation. In the United States, most home care is provided through informal mechanisms by family members [31], and almost 70% of people with SCI receive some form of assistance and support from family members [32]. One individual in a family frequently assumes most or all the responsibilities of caring for a person with a disability [33], and this responsibility can carry with it several physical, emotional, social, and economic risks. We will recruit a sample of 10 SCI veteran family caregivers from each site who have provided care on a daily basis, for at least 6 months, to SCI, and preferably, these family members identify as the *primary* caregivers. Family caregivers who may be eligible to participate in the focus groups or interviews will be identified by clinical staff (social workers, therapists, and others) or an SCI veteran who participated in a semistructured interview. A member of the research team will contact the family caregiver to inform them about the study and determine if they meet the eligibility criteria to provide additional information and make arrangements to obtain informed consent before the start of focus group or interview data collection.

#### Spinal Cord Injury Veteran Clinicians

Clinicians’ perceptions are important because they may affect patient-provider relationships, the course, and the outcome of treatment. Clinicians have knowledge of the medical and functional consequences of SCI and experience providing training to veterans living with SCI and their family caregivers to plan for adjusting to home life and community reintegration. SCI clinicians at each participating center will be informed of the study through written (flyers and emails) and oral communications with local study leaders and/or supervisory staff. We will recruit a sample of 10 clinicians at both sites that includes staff members who have experience providing direct care or services to veterans living with SCI at each of the research sites. The SCI clinical staff will include physiatrists, nurses, social workers, physical, recreation, and occupational therapists who have at least 2 years of experience providing care to SCI. Clinicians who are interested in participating and wish to be contacted by the research team will receive additional information and, if interested, give informed consent before the start of focus group data collection.

Each group of participants has real-world experiences and clinical knowledge that will inform the content of the key practice recommendations that can be readily integrated into clinical guidelines, family interventions, caregiver training, and patient education programs.

#### Study Sites

Two study sites that serve veterans living with SCI in New Jersey will conduct the proposed study: VANJHCS and Northern New Jersey Spinal Cord Injury System. A description of each research site is provided in Multimedia Appendix 1: facilities, existing equipment, and other resources. These sites were selected for their access to diverse communities of veterans living with SCI served by the respective research institutions. Each site has an average of 190 to 429 veterans living with SCI in their respective patient registries.

### Sampling Strategy and Recruitment

After obtaining approval from the IRB at the study sites, purposeful sampling strategies will be used to identify and recruit potential study participants. A research coordinator (RC) will recruit potential participants using advertisements, brochures, and referrals from SCI registries at each collaborative site. If we do not get an adequate recruitment response, we will implement the snowball recruitment technique that involves asking participants to inform and encourage friends, colleagues, and other peers to participate [34].

In recent years, data saturation has become the gold standard by which purposive sample sizes are determined in qualitative research [35]. Theoretical saturation is achieved when focus groups or interviews do not generate novel ideas. The sample sizes proposed for each study phase described below are based on minimum sample size recommendations for common qualitative study designs [36]. Furthermore, our sampling strategy will be flexible, evolving as the study progresses through the 4 phases, until the point of redundancy in emerging themes is reached to meet the purposes of the study.

Once each participant completes oral and written informed consent and is scheduled for interview or focus group session, he/she will receive a telephone reminder 2 weeks before the interview or focus group as well as a written letter 1 week beforehand. The day before the event, the consultant will make 1 last round of confirmatory phone calls. Participants will be compensated for their time. In addition, to maintain enrollment and participation, we will provide transportation cost for a portion of veterans living with SCI and family caregiver on an as-needed basis.

#### Data Collection

Three methods of qualitative data will be collected: chart review, semistructured interviews, and focus groups. These 3 qualitative data collection methods will be implemented over 4 phases of sequential qualitative data collection outlined in Table 1. The content of the chart reviews, interviews, and focus groups will be stratified based on disability benefits. The structure and content of questions will be modified based on joint planning with the community advisory board (CAB) during the course of the project. Of particular interest is how these groups *independently* interpret the reasons and impact of the disability benefits on veterans living with SCIs’ . Results from each phase will be analyzed separately and then merged to inform the content of the subsequent phases as well as the set of practice recommendations that can be readily integrated into clinical guidelines and family interventions. Multimedia Appendix 2 provides detailed description of sample items from the data collection instruments.

**Table 1:** Research plan.

Phase	Data collection	Purpose	Data source
I	Chart review	Prepare and supplement data gathered in subsequent phases	Medical records
II	Semistructured interviews	Exploring SCI^a^ veterans’ understanding and perceptions of VA^b^ disability compensation benefits	SCI
III	interviews/focus groups	Identify SCI family caregivers’ perspectives about the impact of disability benefits on the household	SCI family
IV	Focus groups	SCI clinicians’ perspectives of the impact of VA disability benefits on SCI and provision of adequate long-term health care and rehabilitation	SCI
V	Focus groups	Develop key elements for clinical practice recommendations	Triangulation of study findings, feedback from service

^a^SCI: spinal cord injury.

^b^VA: US Department of Veterans Affairs.

#### Phase I: Chart Review

The RC will be trained to use a standardized chart abstraction instrument to gather demographic, clinical, and disability benefits data. The chart review will be designed to help prepare and supplement data gathered in subsequent phases by providing data that will (1) inform the development of discussion questions for the participant interviews and focus groups; (2) confirm the veterans living with SCI disability status and rating; and (3) documentation of health status, functional information, and patient/family education logs gathered during the most recent annual evaluation of 30 veterans living with SCI (15 per site). An annual evaluation was defined as a *comprehensive annual history/physical exam with specialty assessments*, offering an annual evaluation is mandated for patients with SCI in the Veterans Health Administration (VHA) [17].

#### Phase II: Semistructured Interviews

Semistructured interviews will be aimed at capturing service-connected and nonservice-connected veterans living with SCI perspectives on ways in which their financial compensation (or lack thereof) impacts their health status, functional independence during community reintegration, QOL, and their utilization of health care. The sample will be equally split between study sites (n=30) veterans. We will modify items from the benefits coverage inventory, a measure that has been used in previous research to assess rehabilitation/independent living benefits received after discharge in 5 areas: housing, personal care assistant, transportation; outpatient therapies (eg, physical therapy, occupational therapy, and vocational rehabilitation), and equipment (durable and nondurable). The measure asks about who pays for these items (self, insurance, or other). We will work with the CAB to develop semistructured interview questions that relate to service-connected disability benefits. The semistructured interview will discuss veterans living with SCIs’ likes and dislikes of the being service-connected or nonservice-connected—their perceptions about whether disability benefits may be viewed as a barrier to independence, and difficulties with bureaucracy for some veterans living with SCI. The interview will ask veterans living with SCI to express their experiences about unexpected barriers associated with seeking service-connected financial compensation. Key questions will focus on their health status, maintenance of their functional independence during community reintegration, health care, and rehabilitation experiences. The individual semistructured interview allows for rapport and confidence building at a sensitive time after injury so that more honest opinions and attitudes may be revealed more readily than in a group setting. The interviewer can answer respondent questions, probe for additional answers, and observe visual cues. To facilitate access to veterans living with SCI who are unable to come to the research site for an interview, we will use the VA’s real-time video health tool—Clinical Video Telehealth that is a technology that is frequently used in the VA to promote video communication between patients and providers (see Multimedia Appendix 3—Facilities, Existing Equipment, and Other Resources). To ensure quality data assurance, interviews will be audiotaped and transcribed. After the interview is completed, the RC and research assistant will summarize their notes and review the results with the principal investigator (PI). Spot checks of the transcripts comparing them with the audiotapes will be done to ensure accuracy of the transcripts.

#### Phase III: Spinal Cord Injury Veteran Family Caregivers

SCI family caregivers provide assistance that is critical to sustaining health status, functional gains, and access to health care services as veterans living with SCI return to the community and will provide information about household/community barriers observed across service-connected and nonservice-connected veterans living with SCI. We give family caregivers the option to participate in a caregiver focus groups or individual semistructured interviews. The qualitative data collection methods will be used to ascertain SCI veteran family caregivers’ perspectives on the impact of disability benefits on their family life, including household finances, the health of SCI veteran as well as their own health, and the provision of health care/rehabilitation to sustain the functional independence of the SCI. Caregivers will also be asked to provide suggestions about potential solutions to the problems they identify to facilitate their efforts providing care to an SCI veteran that is service connected or not service connected.

Focus groups are an efficient way to collect data from several people simultaneously, and they explicitly use group interaction as part of the method [34]. Focus groups will allow us to elucidate the shared experience and challenges of seeking disability benefits and the factors associated with outcomes and choice of health care facility among veterans living with SCI with and without service-connected disability benefits. This recruitment strategy will account for nonattendance and ensure optimum focus group size and participant comfort [34]. Multimedia Appendix 2 gives a draft of the focus group script.

The RC will take field notes on a structured data recording sheet, based on the focus of group script/interview guide. The field notes will include key points, notable quotes, and important observations such as silent agreement, body language, group mood, and ironic or contradictory statements. Each focus group will be recorded and transcribed, but anonymity will be maintained. The focus groups will be recorded with a password-enabled digital recorder, and we will transfer all the recordings to the secure VA network after each interview is completed. At the end of each focus group, the PI will give a brief oral summary of critical points that the participants can verify, amend, or change. The PI and RC will meet for a debriefing immediately after each session to share their perceptions of first impressions, critical points, and notable quotes and to highlight and contrast findings from earlier focus groups. This debriefing will also include any notable circumstances that influenced the discussion, resolution of questions, and potential modifications for subsequent groups and/or interviews.

#### Phase IV: Spinal Cord Veterans’ Clinicians

We will conduct focus groups with SCI clinical staff from the Veterans Administration New Jersey Healthcare System and SCIMS health care systems. The focus groups will be conducted with approximately 10 interdisplinary SCI clinicians per group to provide insights into the following: (1) clinical factors of veterans living with SCI who seek different types of disability compensation benefits (ie, service connected and others are nonservice connected); (2) describe their perception about the relationship between disability benefits and health status, functional outcomes and health care utilization among veterans living with SCI; (3) identify VA and private-sector health care system issues related to disability compensation benefits, which are obstacles for veterans living with an SCI; and (4) identify solutions to address these concerns.

#### Phase V: Developing Practice and Policy Recommendations

##### Qualitative Data Analysis Plan

To design a useful set of practice recommendations, we will analyze results from phases I to IV separately and then merge them to prepare the content materials for phase V. The chart review, interview, and focus group data will be prepared for analysis by converting the raw data (eg, field notes) into partially processed data (eg, write-ups and transcripts), which will then be coded and subjected to an analytic theme. The analysis will focus on the key research questions and include the following steps:

1. Read each transcript in an editing style to augment an initial codebook template developed from the ICF guidelines and interview guide.
2. Read and reread highlighted portions to develop keywords (themes, patterns, or categories).
3. Divide the themes, patterns, and categories into groups by the research questions.
4. Examine the convergence/divergence by completing the following steps:

Convergence will be examined by determining what themes fit together to develop the internal homogeneity and external heterogeneity.

Internal homogeneity will be determined by analyzing the themes to see which are more similar, and external heterogeneity was determined by analyzing the themes to see which ones are distinctly different from each other [37].

These processes will enhance the credibility of the research. Divergence will reveal some of the patterns within the categories and helps to make connections among the themes for categorical saturation [37].

Qualitative data collected in phases I to IV will generate its own findings to add additional information to our understanding about the reasons veterans living with SCI seek different types of disability benefits and how these factors that are associated with health status, functional outcomes, and QOL. Triangulation is methodological approach that contributes to the validity and reliability of qualitative data collection when multiple method and sources are employed [38]. Triangulation will allow us to compare, contrast, and integrate the results from the chart reviews, veterans living with SCI, family caregivers, and SCI clinicians. Triangulation from the focus group and interview data will also allow us to ensure results are being confirmed across data sources and identify what is being uniquely provided by different data sources. Investigator triangulation, which consists of multiple—rather than single—observers, will be used to strengthen the validity and credibility of the qualitative findings observed in each phase of the study [38]. A very large amount of raw data will be generated; therefore, narrative data will be stored and analyzed using QSR International's NVivo 12 software. This type of software facilitates thematic coding, interrater reliability, and correlation of themes with demographic characteristics. NVivo can also be used with Excel spreadsheets to generate matrices that demonstrate relationships between variables and themes.

##### Developing Practice and Policy Recommendations

Using the themes generated from the qualitative analyses, we will work with the CAB to summarize to identify the most frequently cited factors (ie, problems and solutions) that impact veterans living with SCIs’ decision to seek service-connected disability benefits (ie, those that are mentioned by more than 1 data source) and across samples of participants—a process that is known as *group-to-group validation* [34].

There are a number of steps outlined in the literature used to develop targeted recommendations for clinical guidelines, developing recommendations for patient and family educational resources, and policy-level interventions. First, the research team will generate a summary of the most frequently cited factors identified from the data as recommendations to the CAB. Examples of potential interventions include revisions to existing patient and family education material related to programs and services available from the VA for veterans with SCI; educational interventions for SCI clinicians to make them more aware of resources available from the VA that are correlated with positive clinical practice outcomes for veterans with SCI veteran, and their families, caregivers, and other clinicians; procedures to help clinicians identify veterans living with SCI at civilian hospitals and connecting these veterans and their families with knowledgeable VA advocates (eg, VA SCI social worker or veteran service organization) and VA disability compensation and health care resources.

Second, the CAB and research team will have monthly consensus meetings, which will be used to evaluate aspects of the most frequently cited individual, family, SCI clinician, and systemic factors generated from the data: importance to veterans living with SCI and modifiability. Determination of importance can be obtained in several ways, such as the CAB rating on the degree of importance, and assessing socially significant implications of the qualitative findings. The CAB and research team will collaboratively rate the modifiability of the individual, family, SCI clinician, and systemic factors from the qualitative data by asking the question, “Can this problem easily be addressed?” or “Can this solution be implemented pragmatically?” The collaborative will rate these individual, family, SCI clinician, and systemic factors as (1) factors that can be completely changed, (2) factors that may be modified, but we are unable to change them completely, and (3) factors that are nonmodifiable. Third, the CAB and research team will select *targets of change*. After identifying the factors that are both important and modifiable, the CAB and research team will decide which factors will be targeted for recommendations for interventions. Finally, the CAB and research team will select promising strategies or key recommendations to develop an intervention action-point document. The intervention action-point document will be a road map toward the operationalization of an educational intervention for SCI, families, SCI clinicians, and policy recommendations for the VBA.

Content of the intervention action-point document will be evaluated assessed for their appeal, clarity, and appropriateness for the target users. We will recruit veterans living with SCI and family caregivers who participated in phases I to III. We will recruit approximately 5 veterans living with SCI and 5 family caregivers. Veterans living with SCI and family caregivers will be asked if they are willing to participate in future groups or interviews. Members of the research team will recruit those who agreed using personal phone calls. If we do not get an adequate response from the previously identified participants, advertisements, brochures, word of mouth, and the aforementioned snowball technique will be used. An honorarium will be offered to compensate participants for their time and transportation.

## Results

The project was funded in July 2015, and recruitment was completed in October 2018.

A total of 69 male veterans living with an SCI participated in medical chart review and interview phases of the study. The mean age of the sample was 59.5 years (SD 14.8; range 23-86). Most of the veterans self-identified as non-Hispanic white (61%, 42/69), married (52%, 36/69), had some college education (80%, 55/69), and unemployed (93%, 64/69). In terms of military experience, approximately one-third served in the army (39%, 27/69) and primarily in the Vietnam era (32%, 22/69). The majority of veterans sustained their injury after military service (75%, 52/69), and were living with paraplegia (53%, 37/69) for an average of mean 15.0 years (SD 13.0. Almost two-thirds; 64%, 44/69) of the veterans self-identified as nonservice connected and 36% (25/69) were service connected for SCI or another disability (eg, PTSD). A total of 18 caregivers participated in focus groups and interviews at the 2 sites. All of the caregivers were females, the mean age of caregivers was 64.8 years (SD 10.8) and reported caregiving for their loved one for approximately, 15.9 years (SD 11.4). A total of 9 SCI clinicians participated in a focus group at Veterans Administration New Jersey Healthcare System and 10 at the SCIMS site. Comparable clinical professions (eg, psychiatrist, nurse, and therapists) were represented in each focus group and average of 11.5 years (SD 8.8) working in their current positions.

Preliminary content review of narrative data suggests that veterans living with an SCI and caregiver participants varied in their reasons why they did not apply for VA disability compensation, including a lack of knowledge and misinterpretations about the VA disability compensation eligibility and health care coverage from the VBA and VHA, respectively.

## Discussion

This qualitative study will use a community-based approach to derive information from veterans living with SCI, family caregivers, and SCI clinicians about their day-to-day experiences with being service connected or nonservice connected status. Of particular interest is how these groups *independently* interpret the reasons and impact of service-connected compensation (or lack thereof) on the health status, functional outcomes, QOL, and health care utilization of veterans living with SCI. Preliminary findings suggest a small proportion of participants receive VA service-connected disability compensation benefits. Participants’ responses indicate that veterans living with an SCI and their caregivers may not be fully aware of their eligibility for VA disability compensation, making it more difficult to make an informed decision about pursuing VA disability compensation benefits. To make an informed decision about eligibility for VA disability compensation benefits, veterans living with SCI should be connected with an experienced veterans benefits advocate (ie veterans service officer). Study findings will be used to generate a set of practice recommendations to the clinical guidelines, family interventions, caregiver training, and patient education programs that can be tested in a future large-scale multisite quantitative study to devise targeted community-based interventions.

## Appendix

Multimedia Appendix 1 Peer-reviewer report from the Department of Defense, United States of America.

Multimedia Appendix 2 Data collection instruments.

Multimedia Appendix 3 Facilities and resources.

## Abbreviations

CAB: community advisory board
ICF: International Classification of Functioning, Disability, and Health
IRB: institutional review board
NNJSCIS: Northern New Jersey Spinal Cord Injury System
NSCID: National Spinal Cord Injury Database
PI: principal investigator
PTSD: posttraumatic stress disorder
QOL: quality of life
RC: research coordinator
SCI: spinal cord injury
SCIMS: Spinal Cord Injury Model System
VA: Department of Veterans Affairs
VANJHCS: East Orange Campus of the Veterans Affairs New Jersey Health Care System
VA SCI/D: Department of Veterans Affairs Spinal Cord Injury and Disorders
VBA: Veterans Benefits Administration
VHA: Veterans Health Administration
