# Supplementary material for: Spinal Cord Injury Veterans’ Disability Benefits, Outcomes, and Health Care Utilization Patterns: Protocol for a Qualitative Study
Source: JMIR Res Protoc. 2019 Oct 4;8(10):e14039. doi: 10.2196/14039 (PMC6800461; doi:10.2196/14039)
Supplement: Multimedia Appendix 1 [file resprot_v8i10e14039_app1.pdf]

DEPARTMENT OF THE ARMY
US ARMY MEDICAL RESEARCH ACQUISITION ACTIVITY
820 CHANDLER STREET
FORT DETRICK MD 21702-5014

REPLY TO
ATTENTION OF

May 18, 2015

MCMR-AAA-A Customer Service Group - Assistance

Denise Fyffe
Kessler Medical Rehabilitation Research & Education Corporation
Department of Spinal Cord Injury Research
1199 Pleasant Valley Way
West Orange, NJ 07052-1424
dfyffe@kesslerfoundation.org

RE: SC140270 - “Spinal Cord Injury Veterans: Disability Benefits, Outcomes, and Health Care Utilization Patterns”

STATUS: RECOMMENDED FOR FUNDING

Dear Denise Fyffe:

Congratulations! On behalf of the Department of Defense (DoD) office of the Congressionally Directed Medical Research Programs (CDMRP), I am pleased to inform you that the Fiscal Year 2014 (FY14) Spinal Cord Injury Research Program (SCIRP) Qualitative Research Award application you submitted was recommended for funding. Funding recommendations were made at a second-tier, programmatic review meeting based on program goals and scientific merit. Please note that awards are subject to negotiations and availability of funds. Additional information on the review process is available in the information paper posted under your eBRAP account.

Additional documentation is needed to start the award process. Your timely submission of all applicable documents in the appropriate formats will expedite this process and the release of funds. A CDMRP Science Officer will be your scientific and technical point of contact throughout the life of this award and may contact you for further information regarding various aspects of your application. For example, your Science Officer will contact you about Animal Use and Human Use documents that may be needed for your project. Together, you will set a target date for the submission of your complete animal use or human use appendix documents. The Surety and Environmental Office will contact you if any Environmental Compliance Assurance documents are necessary.

A Grants/Contract Specialist from the US Army Medical Research Acquisition Activity (USAMRAA) will contact the Business Official (person authorized to conduct negotiations) at your institution to begin award negotiations. All official negotiations of the budget, terms, and conditions of any resulting award will be limited to the Business Official of your institution and the USAMRAA Grants/Contract Specialist.

Your performance on any previous CDMRP awards will be considered during negotiations. As such, you are advised to review your current and past awards to ensure that all required information has been submitted, including all technical reporting and regulatory oversight documents, as this may impact the negotiation and award process.

If you are withdrawing your application, please co-sign a letter of withdrawal with an administrator from the Sponsored Programs Office at your institution and upload it under the "Required Award Information" tab on eBRAP (https://eBRAP.org).

To expedite the award process, please answer the post-submission questions found under the "Required Award Information" tab on the eBRAP website (https://eBRAP.org) by June 2, 2015. For additional information, consult the "Guide for Funded Investigators," which can be found in the "Resources and Reference Material" section under the "Funding Opportunities and Forms" tab on the eBRAP website. Please especially note the following when supplying the information requested under the above-mentioned tab:

- You and your institution are responsible for ensuring that there is no duplication of the science, budget, or level of effort in separately funded studies in which you were or currently are involved. If you received funding for any portion of this application from another source, or if any portion of the proposed work has already started, please indicate so under this tab.
- Updated details on all support for the past 5 years, existing support, and pending support for yourself and key personnel, including the title of the project, goals, specific aims/tasks, estimated start date and end date, level of effort (percentage or calendar months) in the project, and point of contact at the funding agency. Provide a cover letter signed by a sponsored programs official, certifying that this information is current and accurate, and addresses any scientific or financial overlap.

A copy of this notification is being made available to your institution's Sponsored Programs Office.

Again, congratulations on the recommendation of your application for funding. The CDMRP staff and I look forward to working with you to realize the vision of the SCIRP, and we encourage you to share the news of your success with your colleagues and the community. Please direct any questions to the CDMRP Help Desk at help@eBRAP.org or 301-682-5507.

Sincerely,

Elvera M. Messina
Grants Officer

Cc: Linda Bambrick, Ph.D.
SCIRP, Program Manager

Department of Defense
U.S. Army Medical Research and Materiel Command
Congressionally Directed Medical Research Programs
2014 Spinal Cord Injury Research Program
Qualitative Research Award
Peer Review Summary Statement

CDMRP Log Number: SC140270
Grants.gov ID Number: GRANT11773146
Meeting Dates: 12/16/2014-12/16/2014
Review Panel: Qualitative Research

Project Duration: 36 months
Total Budget Requested: $598,000
Direct Costs: $396,027
Indirect Costs: $201,973

Title: Spinal Cord Injury Veterans: Disability Benefits, Outcomes, and Healthcare Utilization Patterns
Principal Investigator: Denise Fyffe
Performing Organization: Kessler Medical Rehabilitation Research & Education Corporation
Contracting Organization: Kessler Medical Rehabilitation Research & Education Corporation

Overview

The Principal Investigator (PI) of this application proposes to address the research problem that there do not appear to be any studies that have compared the impact of having additional financial resources provided to service-connected (SC) veterans with spinal cord injury (SCI) with non-SC veterans with SCI who do not have these additional financial resources using a qualitative approach. The project's specific aims are to (1) describe reasons for veterans with SCI for seeking SC versus non-SC disability compensation and the factors that influence their choice; (2) explore the impact of SC and non-SC disability benefits on health status, functional outcomes, quality of life, and medical decisions (eg, choice of VA SCI Center versus private sector); (3) explore the impact of service connection disability benefits on the family caregivers and households of veterans with SCI; (4) explore SCI clinicians' perspectives of the impact of SC and non-SC veterans with SCI on the provision of adequate long-term health care and rehabilitation; and (5) develop a set of practice and policy recommendations about the impact of SC and non-SC veterans with SCI to clinical and policy guidelines, family interventions, caregiver training, and patient education programs.

	**Average Score**	**Standard Deviation**
**Overall Evaluation**<br>Rating Scale: 1.0 (highest merit) to 5.0 (lowest merit)	2.4<br>**(Good)**	0.2
**Evaluation Criteria**<br>Rating Scale: 10.0 (highest merit) to 1.0 (lowest merit)	**Average Score**	
**Research Problem**	6.0	
**Study Design**	5.3	
**Personnel**	5.3	
**Impact**	6.6	

SCORED EVALUATION CRITERIA

Research Problem
Average Score: 6.0

Scientist Reviewer A

Little is known about the differential impact of SC disability compensation on health status, functional outcomes, quality of life, and health care utilization patterns on veterans with SCI. Overall studies indicate that SC veterans consistently report high rates of medical impairment, psychiatric symptomatology, and utilization of medical and mental health services. These findings indicate that it important to consider a range of factors that could impact veterans with SCI seeking or not seeking SC disability compensation.

Strengths: The major research problem, such as the need to identify the health care utilization patterns and their differential impact of SC and not SC disability compensation on the health status, functional outcomes, and quality of life of veterans with SCI, is clearly stated. The qualitative measures appear to be a reasonable approach to describe the reasons of making health-related decisions. The proposed work is significant because it will provide new knowledge of veterans with SCI, the influence of family caregiver support, access to community resources, and personal factors and health behaviors, including patient-provider relationships and their the impact on SC and non-SC veterans with SCI on health status, functional outcomes, and quality of life.

Weaknesses: The number of those with non-SC, but potentially SC veterans does not appear to be justified. Qualitative versus quantitative approaches do not seem to be discussed.

Scientist Reviewer B

Strengths: The topic is very important, and it addresses a key area of encouragement. The project also takes seriously the community-based participatory approach and thoughtfully uses a panel of experts as a first step to defining the terms of the study and determining final study results. The project combines qualitative methods with some quantitative data collection and uniquely includes study participants beyond individuals with SCI themselves such as clinicians and family members, all of whom bring useful perspectives about the impact of financial resources on outcomes after SCI.

Weaknesses: The 3-phase study design appears to rely entirely on recruitment of n = 15 service- and non-service–related persons with SCI in each site. Although it is reasonable to expect recruitment to succeed, if there are any difficulties then phase 2, the interviews with the person's family caregiver, would be at risk. The application could be strengthened by including additional information stating this potential problem is anticipated and clarifying how the research team would find alternatives to achieve the study goals. Also, while the theoretical model is appropriate to guide the study design and interview questions, the application appears to say nothing about how study results will add to theory. The application reports only how study results will inform practical actions taken by service providers to improve care and education. If

a theoretical model is used, study results should also inform the building of theory; ie, which aspects of the theoretical model are strong, and which may need further development.

Discussion Notes

The panel members had their enthusiasm for the study reduced, believing that SC status is not well described.

Study Design
Average Score: 5.3

Scientist Reviewer A

Strengths: Qualitative data will be collected from chart review, semistructured interviews, and focus groups. Triangulation of these data sources should allow to compare, contrast, and integrate the results into a set of practice and policy recommendations about the impact of SC and non-SC veterans with SCI to clinical and policy guidelines, family interventions, caregiver training, and patient education programs.

Weaknesses: The estimation of sample size (n = 15 for each of groups) does not appear justified based on experience and preliminary data. Inclusion/exclusion criteria for SCI participants do not appear to be specified clinically, socially, and demographically (includes only males). A risk "that the project findings may not be able to solve all of the problems associated with access to comprehensive rehabilitation and medical care for SCI Veterans" is acknowledged; however, anticipated findings and scenarios do not seem to be discussed. All these factors taken together make it difficult to expect strong educational tools as the main outcome from this study.

Scientist Reviewer B

Strengths: The study sites are appropriate and should yield subjects as proposed. The depth of the project, focused on 15 persons with SCI in each of the categories, is appropriate to the topic and will likely hit saturation as the investigators suggest. Disparities in outcomes after SCI are a significant and persistent issue that negatively impacts all dimensions of personal and family life after injury. The study has potential to reveal the characteristics of successful individuals who navigate the barriers to participation and how financial resources make a difference. Knowledge from this study will undoubtedly inform rehabilitation program design and community-based programs and hopefully federal policies as well.

Weaknesses: The qualitative interview questions were somewhat general. More detailed questions that probed deeper questions would provide more valuable information. For example, the application asks about the type of problems that participants have had in obtaining services. However, it appears to fail also to ask about why the subjects think these difficulties occurred and how they believe things could have been different. Perhaps this is intended when the application makes reference to using follow-up questions and probes, but it would be helpful for more of these questions to be specified, so it is clearer exactly what type of information may be gained by doing this study. Finally, there are very limited details about how the qualitative data analysis would be conducted and whether the researchers expect interview questions would

capture differences between family and service members' perspectives and professional providers.

Discussion Notes

After discussion, the panel members generally believed that the number of research subjects planned is not sufficiently substantiated and likely is insufficient. It was also thought that there is insufficient detail on the qualitative analysis, particularly if conflicting information is furnished by different participants. The selection criteria for service-connected subjects were thought to be not sufficiently described. A panel member commented that the importance of SC status for veterans with SCI cannot be overstated. However, SC status is not well described in this application. It is important to remember that Congress makes the rules on eligibility. The sample size appears insufficient, and description of participant selection is weak. This makes it the likelihood of generalizability of the study results weak. Community involvement is good; however, no theoretical model is presented.

Personnel
Average Score: 5.3

Scientist Reviewer A

The PI, Denise Fyffe, PhD, is a clinical psychologist and research scientist in SCI/outcomes and assessment research at the Kessler Foundation in the Northern New Jersey Spinal Cord Injury Model Systems. The PI is also an assistant professor, physical medicine and rehabilitation, New Jersey Medical School, Rutgers, the State University of New Jersey. She received a PhD in clinical/school psychology from Hofstra University in 2000. The research team includes Carol Gibson-Gill, MD, chief of the Spinal Cord Injury/Disorders Service at the Veterans Administration New Jersey Healthcare System (VANJHCS).

Strengths: The PI serves as the site PI at the Kessler Foundation on the multiple collaborative QOL studies in SCI and has facilitated numerous focus groups with patients with SCI, caregivers, and clinicians in the development of SCI-specific quality-of-life measures as well as cognitive interviews with diverse populations. Her work is funded by the Craig Neilsen Foundation. Her record of accomplishments using qualitative methods demonstrates her ability to accomplish the proposed research project. Dr Gibson-Gill has been caring for veterans with SCI for 20 years, and she leads a strong interdisciplinary team and community advisory board (CAB) that will participate in all phases of the design and implementation of the project. The CAB consists of an accomplished group of SC and nonconnected veterans with SCI/D, caregivers, and community advocates.

Weaknesses: The research associate is not named so can not be evaluated.

Scientist Reviewer B

Strengths: The research team has a prior record of successful collaborations and also publications on this topic and related topics.

Procurement Sensitive Document
Do not copy or distribute without CDMRP written permission.

Weaknesses: The research team, together and separately, is engaged in several other funded studies on SCI. They are clearly experts and active, which is positive, but there may be a concern that they do not have sufficient dedicated time for this new study.

Discussion Notes

After discussion, the panel members had significant concerns that although they are highly qualified individuals, their work in other project areas may preclude their spending sufficient time on this project. It also was thought by the panel members to be unclear from the application that there is sufficient expertise in statistical planning and analysis.

Impact
Average Score: 6.6

Scientist Reviewer A

Strengths: The study is proposed to address an area of the FY Spinal Cord Injury Research Program areas of encouragement as a validation of the services provided by VA disability benefits to a potential underutilized source of care and access to care for veterans with SCI. Study findings might be used to generate a set of practice recommendations to the clinical guidelines, family interventions, caregiver training, and patient education programs that can be tested in future large-scale, multisite quantitative study to devise targeted community-based interventions.

Weaknesses: As presented, the potential impact of this study is low because limitations of existing polices and recommendations are not discussed, and expected practical benefits of this study are not listed.

Scientist Reviewer B

Strengths: This study will improve understanding of individuals with SCI who receive more financial resources SC than veterans who are non-SC. Despite the obvious differences in the quality and amount of services and equipment that the SC group receives, the literature does not have strong evidence that these resources are in fact contributing to stronger outcomes. Clinical evidence and logic would suggest these benefits are real but empirical testing is needed. The topic is very much in an area of encouragement. Knowledge gained would be expected to have a direct and immediate impact on all programs providing services to veterans with SCI. The cost of providing care to injured veterans is very high and studies like this are needed to determine what type of financial resources make the most difference to long-term outcomes.

Weaknesses: Overall, the sample size in the study is small. It would be likely that the subjects recruited in the study are not entirely representative of all service members. While this is perhaps fine for the study proposed, since valuable data can perhaps be expected from successful completion of this study, it is still the case that impact would be greater if study results were based on a larger sample.

Procurement Sensitive Document
Do not copy or distribute without CDMRP written permission.

Consumer Reviewer

Strengths: The application is very specific in their approach and aims for SC veterans with SCI versus non-SC connected veterans' benefit status. It addresses the research problems, detailed aims of the research, types of veterans it will help, and how it will assist them. The application addresses a projected timeline for achieving the desired outcome, which is rare in most applications. The potential findings for this project is "real-life" solutions for "real-life" problems, which may possibly provide an independent living and ability to have good health and quality of life. The application has very specific aims for SCI SC versus non-SC benefit status. The application acknowledges potential problems and addresses alternative approaches. The application describes and articulates specific benchmarks to ensure the research is progressing in an efficient and timely manner. The application states how the data will be collected (chart review, semistructured interviews, and focus groups). The research team plans to use triangulation of these data in the impact of the SC and non-SC veterans with SCI.

Weaknesses: No weaknesses were noted.

Discussion Notes

There was concern among the panel members that the small sample size would mean that even if the work is completed as planned, results would risk not being statistically significant and would have limited generalizability.

UNSCORED EVALUATION CRITERIA

Environment

Scientist Reviewer A

The scientific environment is appropriate, adequately supported, and available.

Scientist Reviewer B

No weaknesses are identified in environment.

Budget

Scientist Reviewer A

The budget is appropriate.

Scientist Reviewer B

The budget is appropriate.

Procurement Sensitive Document
Do not copy or distribute without CDMRP written permission.

Application Presentation

Scientist Reviewer A

This application is presented well.

Scientist Reviewer B

The application presentation had no influence on the review.

Procurement Sensitive Document
Do not copy or distribute without CDMRP written permission.
