# Supplementary material for: Spinal Cord Injury Veterans’ Disability Benefits, Outcomes, and Health Care Utilization Patterns: Protocol for a Qualitative Study
Source: JMIR Res Protoc. 2019 Oct 4;8(10):e14039. doi: 10.2196/14039 (PMC6800461; doi:10.2196/14039)
Supplement: Multimedia Appendix 2 [file resprot_v8i9e14039_app2.pdf]

Chart Abstraction Form

Date: __ __ -__ __ -__ __ __ __ Abstractor Initials: ______ Subject ID No: __ __ __

DIRECTIONS: Complete and circle the data applicable to the patient from the chart.

Demographics

1. Gender: M_1 / F_2 **2. Birthdate**: __ __ -__ __ -__ __ __ __ **3. Zipcode:** ________

4. Race: $Black_1$ $Hispanic_2$ $White_3$ $Asian_4$ $Other_5$ $Missing_{88}$

5. Marital Status: $Married_1$ $Single_2$ $Separated_3$ $Divorced_4$ $Widowed_5$ $Missing_{88}$

6. Employment: $Unemployed_1$ Currently $Employed_2$ $Retired_3$ $Missing_{88}$

7. Primary Insurance: None/Self-pay_1 Charity $Care_2$ $Medicaid_3$ $Medicare_4$ $Private_5$ - ___________ N/A_{99}

8. Military Status:

9. Disability Benefits:

10. Medical Diagnoses

10a. Diagnosis # 1: **ICD-9 Code**

10b. Diagnosis #2: **ICD-9 Code**

10c. Diagnosis #3: **ICD-9 Code**

10d.

11. Health Status

Number of hospitalizations in the past 12 months

Length of Stay

12. Functional Status

FIM motor score

13. Social Status

14. Rehabilitation/Medical Goals

Time Review Completed:___________

Total minutes:____________________

Interviewer __ __ __ **Participant ID __ __ __ __ __**

QUALITATIVE STUDY DEMOGRAPHIC FORM

Before we begin the portion of the interview where you respond to questions about your functioning, I will ask you some background questions that will be summarized and used to describe the participants in this study. Remember, your data will be coded by a participant ID number and you will never be identified by name in any analysis.

DRAFT

1. Today's Date __ __/__ __/__ __ __ __
mm dd yyyy

2. What is your age? __ __

3. What is your gender? *(01 = Female, 02 = Male)* ___

4. Have you ever served on active duty in the United States Armed Forces, either in regular military or in National Guard or military reserve unit?

01 = Yes

02 = No

4a. if YES, which war(s) did you serve in:

WWI (01), WWII (02), Korean War (03), Vietnam War (04), Persian Gulf (05); Operation Enduring Freedom (OEF) and Operation Iraqi Freedom (OIF) (06)

4b. if YES to question 4, did you see combat? *(01=Yes, 02=No)* ___

4c. If YES to question 4, what is current military status

01 = Retired from military service

02 = Medically discharged from military service

03 = Discharged from military service

04 = Other: ____ (please specify)

6. In what country were you born? *(01=United States, 02=Other Country)* ____

4a. *[If not born in the United States]* **In what year did you move to the United States?** __ __ __ __

7. What language do you mainly speak at home?

(01) English ___

(02) Spanish ___

(03) Some other language *(specify)* _______________

8. Are you of Hispanic or Latino origin or descent? *(01=Yes, 02=No)* ___

9. What is your race (Mark all that apply)

(01)	American Indian or Alaska Native	___
(02)	Asian	___
(03)	Black or African American	___
(04)	Native Hawaiian or Other Pacific Islander	___
(05)	White or Caucasian	___
(06)	Other *(Specify)* _____________	

10. What is the highest grade in school that you have completed? ___ ___

00 = None	10 = 10th grade
30 = preschool	11 = 11th grade
40 = kindergarten	12 = High School Grad/GED (Preparatoria, Maturita, Abitur, Gimnazium Ogolnokształeace)
01 = 1st grade	
02 = 2nd grade	13 = Some college credit but less than 1 year
03 = 3rd grade	14 = One or more years of college, no degree
04 = 4th grade	15 = Associates degree (e.g., AA, AS)
05 = 5th grade	16 = Bachelor's degree (e.g., BA, AB, BS)
06 = 6th grade	17 = Masters degree (e.g., MA, MS, MEng, MEd, MSW, MBA)
07 = 7th grade	18 = Professional degree (e.g., MD, DDS, DVM, LLB, JD)
08 = 8th grade	19 = Doctorate degree (e.g., PhD, EdD)
09 = 9th grade	

11. Have you ever been diagnosed with an Academic or Learning Disability?

Y (01) N (02) *If yes, explain:* ______________________

12. In general, would you say your health is…?

(01) Excellent ___

(02) Very good ___

(03) Good ___

(04) Fair ___

(05) Poor ___

DRAFT

13. What is your Marital Status? *(circle one)*

Single (01) Married (02) Divorced (03) Widowed (04) Living w/Partner (05)

14. Are you currently employed? *(circle one)* Y (01) N (02)

12a. *IF YES,* title: ______________________ Full-time (01) Part-time (02) Other (03)

15. Are you employed in the same type of work as before your injury? Y (01) N (02) N/A (03)

13a. *IF NO,* list previous occupation:__________________________

16. What is the occupation you have held for the longest time? If you are retired, what is the occupation you held for the longest time before you retired?

01= Professional, Technical, & Related (teacher/professor, nurse, lawyer, physician, engineer)
02= Manager, Administrator, or Proprietor (sales manager, real estate agent, or postmaster)
03= Clerical & Related (secretary, clerk, mail carrier)
04= Sales (salesperson, demonstrator, agent, broker)
05= Service (police, cook, hairdresser)
06= Skilled Crafts & Related (carpenter, repairer, telephone line worker)
07= Equipment or Vehicle Operator & Related (driver, railroad brakeman, sewer worker)
08= Laborer (helper, longshoreman, warehouse worker)
09= Farmer (owner, manager, operator, tenant)
10= Member of the military
11= Homemaker
12= Other, please describe ____
13= Student

17. What was your total household income (income from all sources including child support, alimony, disability, SSI, unemployment) before taxes, in 2010? Please remember your answers are confidential. *(Select One)*

01 = Less than $5,000
02 = $5,000 to $9,999
03 = $10,000 to $19,999
04 = $20,000 to $39,999
05 = $40,000 to $74,999
06 = $75,000 to $99,999
07 = $100,000 or more
08 = Don't know

DRAFT

18. SCI History:

18a. **What is your physician's name?________________ Telephone Number? _______________**

18b. **Have long have you been injured?** _________________ **years**

18c. **What is your level of injury?** ______________

18d. **Is your injury complete or incomplete** INC (01) _____ Comp (02) _____

18e. **What is your AISA classification?** A (01) _____ B (02) ______ C (03) ______ D (04) ______

18f. **Were you injured during:**

Active service (01) ______

Active duty – but on leave (02) _____

After military service (03) ______

18g**. What is your disability rating? ______ (0% to 100%)**

18g. **How were you injured?**

Motor vehicle accident (01) ______

Fall (02) ______

Diving (03) ______

Other Sport(s) (04) ______

Gunshot wound / violence (05) _____

Other (06)____

19. Disability Benefits

19a. Do you currently receive VA disability benefits? *(01=Yes, 02=No)* ___

19b. Do you currently receive VA disability pension? *(01=Yes, 02=No)* ___

19c. Are you considered a catastrophically disabled Veteran? *(01=Yes, 02=No)* ___

19d. Do you receive Department of Defense disability benefits? *(01=Yes, 02=No)* ___

DRAFT

Healthcare

20. In the last 12 months have you received some or all of your health care from VA facilities?

01 = Yes, all of my health care

02 = Yes, some of my health care

03 = No VA health care received

21. Do you have any kind of health care coverage, including health insurance, prepaid plans, such as HMOs or government plans such as Medicare?

01 = Yes

02 = No

22. Was there a time in the past 12 months when you needed to see a doctor but could not because of the cost?

01 = Yes

02 = No

23. Were you ever diagnosed with…

22a. **Depression?**	Y (01) N (02)
22b. **Anxiety Disorder?**	Y (01) N (02)
22c. **Psychiatric Disorder?**	Y (01) N (02)
22d. **Substance Abuse?**	Y (01) N (02)
22e. **Language problems/ difficulties?**	Y (01) N (02)
22f. **Hearing Problems?**	Y (01) N (02)

23. ***Additional Information***

23a. **Approximately how much alcohol do you drink per week?** ________________________

23b. **Do you use drugs/substances regularly, including medications?** Y (01) N (02)

23c. **List of Medications:** ____

DRAFT

Spinal Cord Injury Veterans: Disability Benefits, Outcomes and Healthcare Utilization Patterns

SCI Veteran In-depth Interview Script

Study Objective: To examine the impact of having additional financial resources provided to service-connected SCI Veterans with non-service connected SCI Veterans who do not have additional financial resources. To compare the impact of monetary compensation on the health and functioning, as well as access to assistive devices/resources, medical care and rehabilitation of service connected SCI Veterans to non-service connected SCI Veterans.

Introduction: During this interview, we will be asking questions about your ability to take care of your personal needs (e.g., bathing, getting dressed, bowel and bladder program) and mobility (e.g., transferring). Some of the questions may seem obvious – we are not trying to be callous or insensitive, but rather we are interested in understanding the things that help and get in the way as you try to take care of of your personal needs and getting around to participate in activities that you enjoying doing. We will audio-record this session in order to ensure accuracy in writing up our report. Your responses, however, will not be linked with your name.

Military Experience

1. Were you drafted, or did you enlist? Tell me about that experience *(including dates).*

   (If enlisted, ask what motivated them to join the service.)

2. How old were you when you joined?
3. Had you ever thought about serving in the military before?
4. How long had the war been going on before you entered the military, or did it start after?

<u>SCI injury</u>

1. How long have you been living with a spinal cord injury?
2. How were you injured?

Disability Benefits

You mentioned that you are service-connected (or non-service connected)

1. Why did you seek service-connected or non-service connected disability benefits?
2. What are some of the thing you like about being service-connected (or non-serviced connected)?
3. What do you dislike about being service-connected (or non-service connected)?

<u>Benefits of Rehabilitation Services</u>

1. GENERAL PROBE: Describe your experiences in the rehabilitation hospital that helped to teach you how to take care your daily needs?
   a. What did you learn in your sessions with the occupational therapist that helped you take care of your daily activities? How did the things you learned from your time with the occupational therapist help you when you moved back home?
      i. Did you continue with occupational therapy after inpatient rehabilitation? If so, why? If not, why not?

DRAFT

b. What did you learn in your sessions with the physical therapist that helped you take care of your daily activities? How did the things you learned from your time with the occupational therapist help you when you moved back home?
    i. Did you continue with physical therapy after inpatient rehabilitation? If so, why? If not, why not?

c. What did you learn in your sessions with the social worker that helped you take care of your daily activities? How did the things you learned from your time with the occupational therapist help you when you moved back home?
    i. Do you still see a social worker? If so, why? If not, why not?

d. What did you learn in your sessions with the case manager that helped you take care of your daily activities? How did the things you learned from your time with the occupational therapist help you when you moved back home?

<u>Community Reintegration</u>

Let's talk about your transition to living at home. How did you adjust to home-life after rehabilitation?

a. Your ability to work, or go to school or do other activities outside the home
b. Your participation in leisure activities
c. Your family life

<u>Current Functional Status</u>

1. What type of activities do you need help with?
2. How would you describe your ability to take care of your own personal needs?
    a. What types of challenges do you have with your self-care? (e.g., managing your bowel and bladder programs)

3. How would you describe your ability to transfer or get around (e.g., using your wheelchair)?
    a. What types of challenges do you have with your mobility? (e.g., are you able to move around your home?)

<u>Caregiver</u>

1. Who do assists with taking care of your daily needs?
2. How does your caregiver assist with your daily needs?
3. What are some things that you like/dislike about ways your caregiver assists you in your daily activities?

TRANSITION: Now I would like you to think and share your thoughts about the ways in which other factors can help or get in the way of your ability to take care of your daily activities.

<u>Lifestyle habits</u>

Do you smoke? What do you smoke? Cigarettes? Pot/Weed? How many cigarettes a day? How long have you smoked? How has smoking helped you take care of your daily activities since your SCI?

Do you drink alcohol? If so, how often per day/week? What do you like to drink? How old were you when you started drinking? How has drinking helped you take care of your daily activities since your SCI?

<u>Economic Challenges</u>

Employment: Are you employed? How do your finances get in the way of you achieving daily activities? (e.g., unable to purchase an assistive device?)

Additional Probes – Since having your SCI was ever a time when:

DRAFT

1. You did not have enough money to meet your daily needs? What did you do? Tell me more about that.
2. You did not have enough money to pay your bills? What did you do? Tell me more about that.
3. You did not have enough money for food? What did you do? Tell me more about that.

Medical Insurance/Healthcare:

1. Do you have medical insurance? How does this limit your access to resources that can help your ability to take care of your daily needs?
2. Do you get your outpatient rehabilitation care from the VA? If so, what have you found helpful in the outpatient rehabilitation services that has helped/hindered your ability to take care of your daily needs?

Additional Probes – Since having your SCI was ever a time when:

1. You did not have enough money for your medical care for yourself? What did you do? Tell me more about that.
2. You did not have enough money for your prescriptions? What did you do? Tell me more about that.

Final question: Are there other factors that we have not discussed today that you think help or get in the way of you achieving your daily activities?

DRAFT

Spinal Cord Injury Veterans: Disability Benefits, Outcomes and Healthcare Utilization Patterns

SCI Veteran Caregiver Interview Script

Study Objective: To examine the impact of having additional financial resources provided to service-connected SCI Veterans with non-service connected SCI Veterans who do not have additional financial resources. Due to the high cost of living with an SCI that and its impacts in on the SCI Veteran and their family, these results will help to solve some problems that the family household might have accessing resources, medical care and rehabilitation for their SCI Veteran. SCI Veteran caregivers will help identify the socioeconomic, health risk, caregiving and healthcare barriers that impede their ability to manage the functional needs of SCI Veterans and Non-Veterans. For the non-service connected SCI Veterans and families the results can help to increase their access to rehabilitative care and assistive resources not currently supported by their disability benefits. Caregivers will also be asked to provide suggestions about potential solutions to the problems they identify to facilitate their caregiving.

Introduction: During this interview, we will be asking questions about your assistance in the care of the SCI Veteran with their personal needs (e.g., bathing, getting dressed, bowel and bladder program) and mobility (e.g., transferring). Some of the questions may seem obvious – we are not trying to be callous or insensitive, but rather we are interested in understanding the things that help and get in the way as you try to take care of [NAME SCI Veteran]. We will audio-record this session in order to ensure accuracy in writing up our report. Your responses, however, will not be linked with your name.

1. How long have you been caring for [NAME of SCI Veteran]

2. What types of things do you assist [NAME of SCIVeteran] with? Do you assist with personal needs like the bowel and bladder program? Meals? Bathing?
    a. What types of challenges does [NAME of SCIVeteran] have with self-care? (e.g., managing their bowel and bladder programs)
    b. What types of challenges does [NAME of SCIVeteran] have with mobility? (e.g., ability to move around their home?)

3. Are there some things that [NAME of SCIVeteran] is able to do for themselves? If so, what are they?
    a. [NAME of SCIVeteran] ability to work, or go to school or do other activities outside the home
    b. [NAME of SCIVeteran] participation in leisure activities
    c. [NAME of SCIVeteran] family life

4. What types of things make it difficult for you to care for [NAME of SCIVeteran]?
    a. Are there things that [NAME of SCIVeteran] that make it difficult for you care for their needs?
        i. Health behaviors: smoking or drinking
        ii. Financial resources:
        iii. Disability Benefits: Is [NAME of SCIVeteran] service-connected (or non-service connected?
            1. Why did [NAME of SCIVeteran] you seek service-connected or non-service connected disability benefits?
            2. What are some of the thing you like about being service-connected (or non-serviced connected)?
            3. What do you dislike about being service-connected (or non-service connected)?

DRAFT

iv. Medical care

b. Are there things going on in [NAME of SCIVeteran] home that make it difficult for them to take care of their personal needs?
c. Are there medical issues that make it difficult for [NAME of SCI Veteran] to take care of their personal needs?
d. Are there financial issues that make it difficult for [NAME of SCI Veteran] to take care of their personal needs?

5. Does [NAME of SCI Veteran] have other rehabilitation services that assist with his/her daily activities and personal needs? Physical therapy? Occupational therapy? Case manger or Social Worker?
   a. How do these individuals help [NAME of SCIVeteran] with their personal needs, participation in leisure activities or mobility?

Final question: Are there other factors that we have not discussed today that you think help or get in the way of you achieving your daily activities with the SCI Veterans?

DRAFT

Spinal Cord Injury Veterans: Disability Benefits, Outcomes and Healthcare Utilization Patterns

SCI Veteran Clinician Focus Group Guide

Opening Statement

Thank you for agreeing to participate in this research project. I'm [name] and I will be your facilitator for this discussion. This is [name] who will be your co-facilitator today. During the focus group we will discuss different factors that could contribute to the health and functioning, as well as access to assistive devices/resources, medical care and rehabilitation of service connected SCI Veterans to non-service connected SCI VeteransSCI Veterans. Today our discussion will focus on the functioning of service-connected SCI Veterans and non-service connected SCI Veterans.

In a focus group, it is really important that you express yourself openly. There are no right or wrong answers. We want to know what you think. If you would like to add to an idea, or if you have an idea that is different from someone else's, feel free to jump in. You do not need to wait for me to call on you to talk, but of course only one person should speak at a time. Again, we are pleased that you have taken the time to speak with us today.

We will audio-record this session in order to ensure accuracy in writing up our report. Your responses, however, will not be linked with your name. Also, please be respectful and do not discuss what is said during the focus group with people outside the group.

Because we are recording the focus group, I may remind you to speak up and talk one at a time so that I can hear you clearly when I review the session recordings. Although I am the facilitator, I would like the interaction to flow among you. We only have [time], so I will keep us moving along.

I. Introduction & Ice-Breaker

Let's go around the room and introduce ourselves. Please give your first name or make up a name. You don't have to respond in any order; just feel free to jump in.

Opening question: What are some functional patterns that you observe among SCI Veterans during rehabilitation? During community reintegration are there changes in the patterns? If so, describe – what types of changes do you observe? Why do you think these changes occur? What are some of the factors that get in the way of service-connected SCI Veterans and non-service connected SCI Veterans meeting their functional goals in physical therapy? During rehabilitation? Or outpatient rehabilitation services?

How does disability compensation impact SCI Veterans meeting their medical and rehabilitation goals?

What are some of the factors that get in the way of service-connected SCI Veterans and non-service connected SCI Veterans s meeting their functional goals in occupational therapy? During rehabilitation? Or outpatient rehabilitation services?

What are some of the factors that get in the way of service-connected SCI Veterans and non-service connected SCI Veterans meeting their functional goals in terms of financial needs?

Are you able to provide service-connected SCI Veterans and non-service connected SCI Veterans with effective discharge plans to help them meet their functional goals when they go home? If so, what helps? If not, what hinders your efforts?

DRAFT

What are the factors that get in the way of community and in-home services assisting with community rehabilitation service-connected SCI Veterans and non-service connected SCI Veterans?

What are you recommendations about ways we can help service-connected SCI Veterans and non-service connected SCI Veterans meet and sustain their functioning when they return to their homes?

DRAFT
