# Supplementary material for: Spinal Cord Injury Veterans’ Disability Benefits, Outcomes, and Health Care Utilization Patterns: Protocol for a Qualitative Study
Source: JMIR Res Protoc. 2019 Oct 4;8(10):e14039. doi: 10.2196/14039 (PMC6800461; doi:10.2196/14039)
Supplement: Multimedia Appendix 3 [file resprot_v8i9e14039_app3.pdf]

FACILITIES, EQUIPMENT, AND OTHER RESOURCES

The study team and Kessler Foundation have access to a wealth of resources that will enable the successful conduct of the proposed study. Key aspects of these resources include:

- Ability to recruit from a large population of persons with SCI (>1,500) who receive innovative and high quality care at Kessler Institute for Rehabilitation--one of the most highly respected institutions for SCI rehabilitation in the United States
- A collaboration with the East Orange Campus of the VA New Jersey Health Care System, which will ensure inclusion of veterans in the proposed study
- A well developed rehabilitation research infrastructure that will provide the appropriate administrative support for study implementation
- Ample office and clinical space for meetings and intervention program implementation
- Information and communication technologies that will enable secure data management and facilitate team collaboration

A description of available resources is given in further detail below.

Clinical Resources

Kessler Institute for Rehabilitation (KIR)

Reputation. KIR is an internationally recognized comprehensive rehabilitation system founded in 1948 by the late Henry H. Kessler, MD, PhD. KIR has been recognized for many years as one of the top rehabilitation facilities in the country. Consistent with that reputation, it has been ranked second among the best rehabilitation facilities in the country from 2005-2011 by U.S. News & World Report. In 2010, KIR was a recipient of the prestigious Great Workplace Award from The Gallup Organization. It honors organizations, only 25 world-wide, whose employee engagement results demonstrate they have outstandingly productive and engaged workforces. KIR is certified by the Joint Commission and has a three-year accreditation from the Commission on Accreditation of Rehabilitation Facilities (CARF) for its comprehensive inpatient rehabilitation programs and subspecialties in the inpatient and outpatient SCI, brain injury, stroke, and amputee programs.

Size and diversity of population served. KIR and the Kessler Foundation collaborate closely to form the Northern New Jersey Spinal Cord Injury System (NNJSCIS), one of 14 SCI Model System centers recognized by the National Institute on Disability and Rehabilitation Research (NIDRR). NNJSCIS facilities serve a densely and heavily populated catchment area in the midst of a highly urbanized and growing geographic region. The majority of New Jersey residents are situated in the 13-county catchment area of the NNJSCIS, which has a population of 6.6 million people; one-third of this population (i.e., over two million people) are racial and ethnicity minorities and 52% are women. All are within a 50-mile radius of a KIR facility. The population density of the areas surrounding our facilities enables the NNJSCIS to reach a large number of persons with SCI with our clinical rehabilitation services and consumer programs. An average of 420 individuals with traumatic and non-traumatic SCI are admitted per year and an estimated 1,500 outpatients with SCI are treated annually.

Facilities. The SCI Service is housed primarily at KIR's West Orange facility with 24-hour, in-house physician coverage and rehabilitation nursing services. This facility has 152 inpatient beds that are housed in a new 100,000 square feet, 3-story building completed in 2006. The SCI unit occupies one entire floor (~40,700 square feet) with 48 beds. This unit encompasses up to 12 ventilator dependent beds and designated beds for patients with dual diagnoses (e.g., concomitant traumatic brain

injury [TBI] and SCI). The SCI unit has state-of-the-art amenities including in-line oxygen, accessible showers in every room, and environmental control unit adaptors in the headboard panel of every bed. The in-patient SCI therapy gyms comprises 9,400 square feet, with two different body-weight supported ambulation systems, multiple electrical stimulation bicycles with arm ergometry, and a complete electronic aids to daily living suite. KIR also houses a 3,700 square foot SCI-specific outpatient gym, outfitted with equipment especially designed for persons with SCI (Lite-Gait®, Thera-Stride®, and Lokomat® Pro body weight support systems, electrical stimulation bicycle ergometers, UE and LE Bioness; WalkAide; dynamic and adaptive UE splinting; Saeboflex; adaptive equipment for ADL and adaptive cardiovascular and strengthening equipment. The SCI outpatient medical clinic includes 11,046 square feet of ambulatory care space with two urodynamic-cystoscopy suites (one equipped with x-ray equipment and fluoroscopy), 13 large examination rooms, and a full electrodiagnostic laboratory. It is open daily to deal with acute and urgent problems as they arise, as well as to provide routine follow-up at 1-,3-,6-, and 12-months post discharge and annually thereafter. A full-time rehabilitation aide is present in the clinic to assist with transfers and care. All KIR facilities are accessible to people with disabilities and are in compliance with section 504 of the Rehabilitation Act of 1973 and the ADA.

Comprehensive SCI Programs and Services. NNJSCIS provides a full spectrum of care from emergency and trauma services to acute care, inpatient rehabilitation, outpatient services, community reintegration services, and lifetime consumer and family support. This is accomplished by means of collaboration with University Hospital in Newark, NJ, KIR, and Kessler Foundation, all of which conduct programs, services, and research to improve the lives of persons with disabilities living in the community.

The KIR SCI Program is directed by Steven Kirshblum, MD (NNJSCIS Co- Director with Dr. Trevor Dyson-Hudson). KIR has nine experienced board certified physiatrists who are also board certified in SCI Medicine. Medical specialists in internal medicine, pulmonology, urology, and other disciplines provide inpatient and outpatient care to persons with SCI. Inpatient and outpatient rehabilitation is provided by an interdisciplinary team that includes physical therapy, occupational therapy, social services/case management, vocational services, education, psychology/neuropsychology, therapeutic recreation, nutrition, pharmacy, respiratory therapy, speech pathology, and prosthetics/orthotics. Patients and family members are also considered important members of the rehabilitation team. Specialized wheelchair and adaptive seating services are provided by nine Assistive Technology Professionals (ATP) who are certified by the Rehabilitation Engineering and Assistive Technology Society of North America (RESNA). SCI nurses are accessible 24-hours a day by phone in order to provide outpatients with information and referrals to the SCI physician on-call if needed.

Specialized programs are offered in the areas ventilator care, wound care, substance abuse counseling, dual diagnosis (SCI and traumatic brain injury) rehabilitation, spirituality, peer counseling and support, career guidance, impaired vision, adapted driving, accessible transportation, adapted sports and recreation, canine-assisted therapy, and womens’ care. See the Table below for highlights of unique services and programs offered through the NNJSCIS.

**Table 1. Summary of Unique NNJSCIS Service Comprehensiveness**	
Prevention	• *ThinkFirst* Program sponsorship with local, regional, and national training*<br>• Public service announcements in prevention of injury*<br>• Involvement with local EMS programs in SCI training*
Acute	• Physiatrist as part of the trauma team<br>• Non-invasive respiratory management in acute setting after SCI
Rehabilitation	• Nine Board certified physicians in SCI Medicine<br>• Specialized "dual diagnosis" and ventilator dependent programs<br>• Comprehensive rehab team including a pharmacist and nutritionist<br>• Ventilator training program for all staff working on SCI floor<br>• Comprehensive Urology program with a triple boarded urologist as Director<br>• Sexuality and fertility program directed by an SCI specialist*<br>• Women's Health services directed by an Ob-Gyn*<br>• Art therapist as part of the SCI Therapeutic Recreation team<br>• Travel program sponsored at Newark International Airport & Continental Airlines*<br>• Peer Counseling Program with specific training modules
Follow up Care	• One of seven Neurorecovery Network centers and a locomotor training site<br>• Wheelchair/Seating Program that includes many full-time RESNA certified therapists<br>• Large diagnosis-specific SCI outpatient gym with Wellness Program*<br>• Innovative surgical interventions that include being one of the first centers to perform diaphragmatic pacing and the implantation of nerves into the myocutaneous flap<br>• Technology leader, most recently in new Lokomat, Ekso, and ReWalk interventions
Vocational and Community Integration Services	• Full time vocational counselor on staff *<br>• Funding vocational programs that create employment for persons with SCI*<br>• Sponsorship of recreational programs including *WheelBlazers*, competition training & travel, and local events (i.e. annual Memorial Day race) *<br>• Training students SCI care including medical, nursing, PT and OT programs *<br>• Training residents from Rutgers New Jersey Medical School as well as other programs*<br>• Involvement and sponsorship of Community Programs that involve persons with SCI*

* Denotes NNJSCIS activities coordinated with services provided by other community resources

KIR has a well-developed education program for persons with SCI, which will be adapted to create the Health and Function education series to be provided to the control group in the proposed study. A CD entitled *Introduction to SCI*, produced by KIR, is given to families to help them learn about the basic anatomy, physiology, and medical issues associated with SCI. An inpatient didactic series for patients is held two days each week to help patients and their families understand the effects of SCI on the body, to encourage self-advocacy, and to prepare them for transition into the community. Patients are provided an SCI manual (available in English and Spanish) that serves as a reference for the series. An

evening education series is also held for inpatients, outpatients and families to allow reinforcement of the daytime program and provide detailed information about SCI on issues ranging from medical

**Table 2. Patient and Family Education Programs and Materials**	
Daytime Education Series Topics	
**How SCI Affects Your Body**<br>• NLI – ASIA<br>• Bowel Management<br>• Bladder Management<br>• Skin Care<br>• Respiratory Care	**Staying Healthy after SCI**<br>• Nutrition and Weight<br>• Exercise<br>• Aging<br>• Medications<br>• Alcohol and Substance Abuse
**Medical Complications after SCI**<br>• Pressure Ulcers<br>• Dysreflexia<br>• Bowel Impactions<br>• Bladder Infections<br>• Neurogenic Pain<br>• Heterotopic Ossification	**Restructuring your Life after SCI**<br>• Attendant Management<br>• Your Rights<br>• Disasters<br>• Sex and Reproduction
Evening SCI Family/Patient Education Series Topics	
**Week 1: Dr. Lam**<br>• How SCI Affects your Body<br>• NLI<br>• Medical Complications	**Week 2: Urology Services**<br>• Bladder Management
**Week 3: Dr. Brooks and Dietician**<br>• Bowel Management and Nutrition	**Week 4: Sandy DeLeon RN**<br>• Skin Care & Pressure Ulcers
**Week 5: OT Staff**<br>• Home Modifications	**Week 6: PT staff**<br>• Advanced SCI Therapies
**Week 7: Outpatient therapy staff**<br>• Out-Patient Therapies	**Week 8: Vocational Counselor and Case Management**<br>• Employment & Vocational Rehabilitation<br>• Discharge Planning & Case Management
**Week 9: Recreational Therapy**<br>• Recreation	**Week 10: Dr. Kirshblum**<br>• SCI Research
**Week 11: Dr Kirshblum and Bonnie Evans (CEO)**<br>• Open Forum	

complications through community integration. In addition to specifics regarding medical and rehabilitation care, educational topics include personal assistance services and management, employment, and recognizing and reporting abusive conditions. (See Table 2 above for a recent schedule of education classes provided in the education series.) All patients receive a Resource Backpack at admission with materials in English and Spanish (See Table 3 below), with some available on audiotape. Ongoing education is also provided through monthly New Jersey Outreach

support groups which features guest speakers on issues including adaptive sports/recreation and financial planning. Additional SCI consumer education is provided via our *Connections* newsletter, the NNJSCIS website, and on our Facebook page.

The NNJSCIS is also committed to the education of health care professionals. Members of other centers, local, national, and international, including physicians, nurses, and therapists, regularly come to KIR for training in SCI care. Examples include serving as a training site for ventilator management (weaning protocols, use of manual in/exsuflation) and for the NeuroRecovery Network (NRN). Our DVD, *Sexuality Reborn***,** is used at many centers around the US and the world. KIR also hosts the national Physical Medicine and Rehabilitaion review course for physicians and the Certified Rehabilitation Registered Nurse course, which are well attended.

Community Outreach. NNJSCIS community outreach emphasizes the psychosocial and recreational needs of consumers who are encouraged to participate in ongoing recreational programs sponsored by the KIR and KF (i.e. *Sports on Wheels*, scuba diving, sailing, arts programs, and wheelchair racing described above). Information about these activities is disseminated via our SCI newsletter, *New Jersey SCI Outreach*, the NNJSCIS Facebook page as well as through event fliers and advertising. The NNJSCIS institutions have also partnered with outside agencies such as the United Spinal Association, Christopher and Dana Reeve Foundation, and the Francis Black Healthcare Foundation to sponsor recreational events.

Table 3. Selected Backpack Educational Materials

Resource/Guide/DVD	***Publishing Organization***
New Jersey Resources 2011	NJ Dept of Human Services, Division of Disability Services
Paralysis Resource Guide	Christopher and Dana Reeve Foundation
Peer Profile Directory: From the Voices of Spinal Cord Injury	KIR
"Yes, You Can!"	PVA Manual
"Guide to Handicapped Parking"	New Jersey Booklet
Children's book entitled *I Will Teach You Everything You Need to Know*	Steven Kirshblum MD. Christopher and Dana Reeve Foundation
"SCI Life"	The National Newspaper of The National SCI Association
"New Mobility"	No Limits Communications, Inc.
"NJ Workability"	NJ Dept of Human Services, Division of Disability Services
Christopher Reeve: Hope in Motion	Arts Alliance America
Tuesday Evening Spinal Cord Series 2011	KIR
SCI/Recreation & Peer Program Monthly Support Group Meeting Schedule	KIR
Business Cards for Education Staff	KIR

East Orange Campus of the VA New Jersey Health Care System

The Department of Veterans Affairs New Jersey Health Care System (VANJHCS) is a consolidated facility comprised of two main campuses, one in East Orange and one in Lyons, 22 miles to the west of the East Orange Campus. The East Orange Campus of the VANJHCS is designated as a

Veterans Affairs Spinal Cord Injury Center, and provides inpatient and outpatient services to veterans with spinal cord injury.

A satellite outpatient clinic is located in Brick, and access points are located in Elizabeth, Fort Monmouth, Hackensack, Jersey City, Morristown, Newark, New Brunswick, Paterson, and Trenton. Primary care is emphasized at the VANJHCS. Veterans are assigned their own health care providers whom they see on a regular basis. Access to a wide variety of specialists is available through the primary providers. In addition to general medical, psychiatry, and long-term care, a full range of medical and surgical subspecialty care is provided to veterans of the VANJHCS in a variety of special programs as listed below. Because the VANJHCS is dedicated to developing programs that meet all veterans' needs, teams of physicians, nurses, therapists, social workers and others all work together to develop programs of distinction that often become models for the health care field.

The System's Diabetes Education, Pulmonary Rehabilitation, Prosthetics, Homeless Outreach Programs, and others have been recognized for outstanding achievement both within the Department of Veterans Affairs and the general health care community. A recognized leader in research, the VANJHCS maintains a very active research program with over 60 investigators conducting pioneering work in areas such as infectious disease, cardiovascular disease, and the health effects of smoking. The East Orange Campus was selected as one of two new research centers that are looking at ways to care for active duty patients and veterans with war-related illnesses.

Special programs available at the VANJHCS (East Orange and/or Lyons Campuses) include Agent Orange, Allergy/Immunology Program, Audiology and Speech Pathology, Cancer Screening, Cardiac Catheterization Lab, CAT Scan, Comprehensive Integrated Inpatient Rehabilitation Program (CIIRP), Day Treatment, Diabetes Education, Healthy Aging and Recovery Care Program (HARP), Hemodialysis Program, Home Based Primary Care, Homeless/Domiciliary Program, Infectious Diseases Unit, Linear Accelerator, Low Vision Center, Magnetic Resonance Imaging, Methadone Maintenance, Nuclear Medicine Unit, Nursing Home Care, Pain Management Program, Persian Gulf Family Support Program, Post-Traumatic Stress Disorder (PTSD) Unit, Primary Care Program. Prosthetics Pulmonary Function Lab, Radiation Therapy, Rehabilitation Programs, Renal Program, Same Day Surgery, Sexual Dysfunction Unit, Sleep Studies Laboratory, Spinal Cord Injury Unit, Substance Abuse Treatment Unit (SATU), Swallowing Program, War-Related Illnesses and Injuries Study Center, and Women’s Health Services.

Virtual Medicine Technologies:

The virtual health technology program is run by the SCI/D Telehealth RN Coordinator who works closely with the veterans, providers and veterans’ caregivers to set up the clinic visits using these technologies. Interviews will be conducted at the facility and in the veterans’ homes.

This VA’s SCI/D Center has had success with using Virtual Medicine technologies since 2000 as a way to decrease the costs associated with the travel to our SCI/D Center, decrease inconvenience to the patients and family, and decrease possible interruption to skin integrity from prolonged time sitting up during the travel process. Virtual Medicine also makes it easier to make changes in the treatment plan without having to bring the patient all the way to the SCI/D Center, helps promote continuity of care for the veteran by the SCI clinician examining the veteran while the Visiting Nurse Agency staff is in the home to discuss the case with the agency staff, as well as discuss treatment options and observing how the treatment is being implemented by the staff and/or caregiver (and patient). We use it to provide a broad range of assessments (e.g., equipment, wounds, etc), education, nutritional support, pharmacy support, psychosocial support and intervention, spiritual care and integrative medicine interventions to our patients and their caregivers (hired and nonhired) in the home.

With **secure messaging** (a secured method of communication with healthcare providers via e-mail), patients can communicate directly with health care workers about their condition and /or needs. This saves them from trying to track down their providers by phones. They usually get a response within 3 business days.

With video conferencing to home, providers and patients can have clinic visits with the providers being at the facility while the patient is in his/her home. This allows the health care team to not only address the patient's concerns but also those of their caregivers (both skilled and unskilled).

Technologies used:

ATI Patient and Provider units allow the provider to not only visualize the patients but to listen to the veterans hearts, lungs, etc through a stethoscope, take his vitals (except temperature), etc.

The videophone system: About the size of a commercial telephone set with a very small video screen. Two videophones are required: one at each the patient's home and the VA clinic room. This system utilizes a plain old telephone service (POTS) line and provides video and audio. It provides a tool to visualize the wound and the care setting and to incorporate the patient's home caregivers, i.e., family, nurse, home health aide, into the treatment process.

Telehealth / Health Buddy system: This system utilizes disease management protocols (DMPs) which are interactive program dialogues tailored to the patient's responses. DMP dialogues include questions that the Vet answers on an in-home device that are then transmitted to a service that is accessible to the provider online. This service will alert the provider if there is an issue that deserves attention. Questions are directed at Vet's self-assessment of wounds, skin, and issues associated with spinal cord injury or disorders. The DMP is designed to assess for conditions that need attention and assess for knowledge deficits then offer targeted educational dialogues. Examples of some of the topics for education might be: monitoring skin integrity, pressure relieving techniques, wheelchair safety and proper cushion adjustment, bowel and bladder management.

MOVI : This allows many of the same benefits as the older videophone but with a higher quality more reliable connection. Patients who have recently been discharged to home after surgery (including surgical repair of pressure ulcers) are being monitored via MOVI as well. The high quality and reliability of MOVI enables video visits with multiple members of the interdisciplinary team in the same appointment that might include: physician, nurse practitioner, social worker, nutritionist, psychologist, wound care nurse, physical medicine and rehabilitation service, Neuro-urologist, SCI/D Center nursing staff, therapist, plastic surgeon, etc.

Clinical Video Telehealth (CVT): CVT is a technology that is frequently used to promote video communication between two or more clinical sites: one at the location where the patient and a provider are and the other at the location with the SCI/D specialists. This is high quality reliable audio & video communication with the ability of the specialist to control the video camera, i.e., zoom in/out, rotate, etc., at the remote site where the patient is located.

Research Resources – Kessler Foundation (KF)

Mission and Scope

KFRC is the research division of the Kessler Foundation, Inc., a 501c3 non-profit public charity. It is a premier medical research organization conducting rehabilitation research designed to improve health, increase function, and promote wellness for persons with physical and cognitive

disabilities. KFRC offers research, training, and educational programming for professionals and consumers in an accessible environment and disseminates research findings to a broad audience of clinicians, researchers, administrators, and policymakers. KFRC houses six specialized research laboratories: Spinal Cord Injury Research, Human Performance & Engineering Research, Neuropsychology & Neuroscience, Outcomes & Assessment Research, Stroke Rehabilitation Research, and Traumatic Brain Injury Research.

KFRC has a full-time staff of 81 individuals, with an annual operating budget of approximately $10.9 million. There are formal affiliation agreements with KIR, Rutgers New Jersey Medical School and the James J. Peters VA Medical Center (Bronx, NY). Other important local collaborators include the New Jersey Institute of Technology, Rutgers University and the Department of Veterans Affairs New Jersey Health Care System in East Orange, NJ. As a NIDRR-supported Model System for both SCI and TBI, KFRC has research collaborations with all of the funded centers. KFRC is accredited by the Accreditation Council for Continuing Medical Education (ACCME) and has conducted more than 25 continuing education courses. One of these courses is the internationally renowned Annual PM&R Board Review Course that has prepared physicians for board certification since 1989.

Research and Education Facilities

KFRC has two locations. The main facility, located at KIR in West Orange, NJ, includes ~20,000 square feet of research and education space and encompasses laboratories supporting research in SCI, Outcomes and Assessment, Human Performance and Movement Analysis, Traumatic Brain Injury, Stroke, and Rehabilitation Engineering and Analysis. Education facilities at KFRC include a state-of-the-art conference center [Kessler Conference Center] (3,150 square feet with a seating capacity of 260) that can be subdivided into three individual teaching areas, each with a seating capacity of 65. The Center is fully wheelchair accessible and can accommodate up to 125 wheelchair users safely. It has its own parking lot with 25 van-accessible parking spaces and overflow parking into the adjacent hospital parking lot when more accessible parking spaces are needed (e.g. for SCI consumer conferences). Our facilities include a separate, smaller conference room (1,152 square feet with a seating capacity of 80), and several meeting rooms appropriate for small group sessions. The second facility, located 1 mile from KIR, encompasses 17,000 square feet and houses the Neuropsychology and Neuroscience Laboratory, additional administrative office space, and the Kessler Foundation Program Center. All KFRC facilities are accessible to all people with disabilities, equipped with adaptive technology for consumers and employees with disabilities, and are in compliance with section 504 of the Rehabilitation Act of 1973 and with the ADA. Many KFRC offices are wide enough to accommodate power wheelchairs and contain raised desks and adaptive computer equipment for increased utility by persons with limited motor function. Working conditions are further optimized by accessible shared spaces (i.e., bathrooms and meeting rooms) and workday flexibility that ensures the full inclusion and success of employees with disabilities.

SCI Research Program

Mission and Scope. The SCI Research Laboratory conducts interdisciplinary research to prevent the loss of function, restore lost functions, and eliminate secondary health conditions following SCI. The ultimate goal of our research is to improve the quality of life (QOL) of individuals with SCI. The SCI Research Lab is led by Co-Directors, Trevor Dyson-Hudson, MD, and Steven Kirshblum, MD, and is staffed by three research scientists, one research coordinator, two nurse coordinators, one pre-doctoral fellow, four full-time research assistants, and full-time secretarial support. At the center of this multidisciplinary laboratory is NNJSCIS research led by Drs. Dyson-Hudson and Kirshblum. The SCI Lab, collaborating with KIR and Rutgers New Jersey Medical School (formerly part of the

University of Medicine and Dentistry of New Jersey), has been a highly successful, NIDRR-supported SCIMS center since 1990. It has contributed extensively to the National Spinal Cord Injury Database and conducted many important research studies regarding community integration, accessibility, and the prevention and treatment of secondary health conditions such as pain, upper limb overuse syndromes, urinary tract infections, cardiovascular disease, and respiratory complications. The lab has also focused on the development of an SCI-specific measure of health-related QOL for use in clinical trials, something that has been missing from current clinical trials involving persons with SCI, and research utilizing Virtual Reality (VR) technology to improve driver retraining and the community integration of persons with SCI. We have recently expanded our research into the area of health disparities in outcomes for persons with SCI. Research in the SCI Lab has been funded by many sources including NIDRR, National Institutes of Health (NIH), Craig H. Neilsen Foundation, New Jersey Commission for Spinal Cord Research, Christopher and Dana Reeve Foundation, United Spinal Association, and industry (i.e., Aventis Corp., Pfizer Corp., Acorda Therapeutics, Coloplast, and Proneuron Corp.).

Extensive History of Collaboration. The partnering NNJSCIS institutions have a lengthy history of local, national, and international collaborations that have fostered a productive track record in the completion of large-scale clinical trials, surveys, and technologically advanced research studies. Collaborative studies are funded across a range of agencies, including philanthropic organizations (e.g. Craig H. Neilsen Foundation); industry (e.g. Coloplast); federal government (e.g. NIDRR, CDC); state government (e.g. New Jersey Commission) and Veterans Administration (e.g. Center Grant).

Locally, the NNJSCIS combines the expertise and scope of a premiere rehabilitation research institution (KFRC) and nationally recognized rehabilitation hospital (KIR) that are aligned with the acute care and academic resources of Rutgers New Jersey Medical School. These cooperative institutions have formal, longstanding affiliations with interlocking professional staff at the levels of middle and top management, thus uniquely positioning the NNJSCIS to continue its tradition of collaborative work. KFRC's research is greatly facilitated by the excellent clinical services and staff at KIR, which is very supportive of research, training, and education. Furthermore, KIR has strong ties to an extensive community of SCI patients, caregivers, and advocates throughout New Jersey. The SCI Lab at KFRC and the main KIR facility share a building in West Orange, NJ. KFRC scientists, KIR physicians, and psychologists have faculty appointments at Rutgers New Jersey Medical School in the Department of Physical Medicine and Rehabilitation and contribute to the education of residents, fellows, and postdoctoral students. Extensive collaboration also occurs within the laboratories that comprise the KFRC and with the Kessler Foundation's Program Center, which improves the lives of persons with disabilities through grant making, community outreach, education, and political advocacy for employment equality.

The external collaborative efforts of the NNJSCIS encompass longstanding relationships between our center and other SCIMS centers, other state and federally funded institutions, philanthropic organizations, the Veterans Administration (VA), and industry. Such efforts include:

- <u>Collaboration on SCI Model System Research Projects.</u> Throughout its 20-year history, the NNJSCIS has expanded their collaborative capacity by leading and supporting numerous collaborative projects conducted by the Spinal Cord Injury Model Systems (SCIMS). A recent project, *Prevention of Respiratory Complications after Spinal Cord Injury: A Randomized Controlled Trial,* involved a partnership between Rutgers New Jersey Medical School, KIR and KFRC as well as the James J. Peters VA Medical Center, Bronx, NY. Over 160 persons with SCI were screened for this project and 121 were enrolled. Another recent multi-site study, *Measuring Quality of Life in Spinal Cord Injury: The Next Generation of Instruments (SCI-CAT),* was jointly led by NNJSCIS and the New England Regional SCI Model System at

Boston University, and was supported by our SCIMS colleagues at Rehabilitation Institute of Chicago, University of Michigan, Mount Sinai Hospital in New York, Craig Hospital in Colorado, and Santa Clara Valley Medical Center. The NNJSCIS led all centers in participant recruitment for this project, having collected approximately 25% of the sample (N=855). The generalizability of the study results have been extended to address other clinical issues in SCI populations including pediatrics (Shriners Children's Hospital), Polytrauma (Tampa Bay VA), and blood pressure control in SCI (Bronx VA). The NNJSCIS also participated in and provided in-kind support for the SCIMS collaborative module project, *Assistive Technology for Mobility*, led by the University of Pittsburgh (see M. Boninger, Letter of Support) as well as a NIDRR Field Initiated project, *Developing a Relevant Instrument to Assess Caregiver Distress and Benefit in Spinal Cord Injury* led by Craig Hospital. NNJSCIS also recently participated in two of the three Multi-Center, Collaborative Projects in SCI funded by NIDRR: the *Collaboration on Upper Limb Pain in Spinal Cord Injury (CULP-SCI)* led by the University of Pittsburgh; and the *Pharmacological Management of Dyslipidemia and Cardiovascular Disease in Persons with Chronic Cervical Spinal Cord Injury* led by the University of Miami.

- <u>Participation in other National and International Multi-Center Studies</u>. The NNJSCIS is one of seven participating centers in the co-operative and coordinated network of the *Christopher and Dana Reeve NeuroRecovery Network (CDRF-NRN)*. As a leading NRN recruitment site, we have delivered more than 1000 intensive, activity-based rehabilitation treatments such as locomotor training to more than 70 people with SCI. NNJSCIS researchers also have extensive experience in collaborating on industry-sponsored multi-center clinical trials in SCI (e.g. Fampridine-SR [Acorda Therapeutics], ProCord [Proneuron], and SpeediCath [Coloplast]).

- <u>Partnership with the James J. Peters VA Medical Center</u>. KFRC and KIR have a joint, formal affiliation with the Department of Veterans Affairs Rehabilitation Research and Development (VA RR&D) Center of Excellence on the Medical Consequences of Spinal Cord Injury (COE-MCSCI) located at the James J. Peters VA Medical Center (JJPVAMC), Bronx, NY. A JJPVAMC satellite research laboratory has been active on the KIR campus since 1998 under the supervision of Drs. William Bauman, (JJPVAMC) and Kirshblum, (KIR). This satellite unit conducts clinical research in the areas of metabolic, pulmonary, autonomic, and cognitive functioning of persons with SCI. It has contributed significantly to research in SCI Medicine, having jointly published 14 peer reviewed articles since 2005.

Our experience in successfully recruiting participants for the national SCIMS database and our SCIMS-related research projects, as well as the success in our collaborative projects over the years as listed above, demonstrates the considerable access to research participants and the infrastructure and culture of cooperation that are hallmarks of the NNJSCIS. We have consistently maintained data quality for our projects and adhered to the research standards for human subjects research as set by our Institutional Review Board as well as by the Institutional Review Boards of our collaborative partners. In sum, the NNJSCIS is committed to continuing our long and successful tradition of collaboration, which in turn will ensure the continued success of our research program.

SCI Publications and Presentations. NNJSCIS personnel have contributed to most of the Consortium Guidelines published by the Paralyzed Veterans of America in the roles of primary writer, reviewer, and chair of the Consortium Committee. Our personnel have written monographs on the equipment used in SCI and on the neurogenic bladder. We have authored numerous chapters in textbooks on SCI, as well as two textbooks covering the field of SCI (*Spinal Cord Medicine* and *Spinal*

Cord Injuries: Management and Rehabilitation). NNJSCIS key personnel have contributed to more than 100 publications and 200 presentations since 1990.

Consumer Involvement in SCI Research. Over the past 20 years, the NNJSCIS has been committed to consumer involvement in the development and management of its research projects. The NNJSCIS has a well-established, active SCI Community Advisory Board, chaired by Dr. Trevor Dyson-Hudson, NNJSCIS Project Co-Director and Director of SCI Research at the KFRC. Dr. Dyson-Hudson is responsible for ensuring the participation of individuals with disabilities in all aspects of NNJSCIS research. As a researcher, physician, and an individual with SCI, his unique perspective continues to foster close collaboration and understanding between members of the SCI community and NNJSCIS investigators. The SCI Community Advisory Board consists of a diverse group of informed and committed persons with SCI, their family members, and SCI advocates. The NNJSCIS research staff, SCI Community Advisory Board, as well as our commitment to Participatory Action Research and its emphasis on consumer involvement in every aspect of research, ensure that we will continue to conduct research that is meaningful in the daily lives of persons with SCI.

Office of Grants Administration (OGA)

Kessler Foundation's OGA provides support for researchers in securing external support for sponsored projects and collaborations. The office reviews, negotiates, and provides administrative oversight related to proposals and the implementation of awards on behalf of Kessler Foundation, in compliance with all Federal, State, sponsor, and internal policies and regulations. The OGA is the central point of coordination for sponsored projects and, in conjunction with the President's office, is the authorized representative for grants, contracts, and other agreements with government agencies, private industry, and non-profit foundations. The OGA works closely with the Finance Office on all fiscal matters of KFRC grants and contract accounts to ensure that funds are spent appropriately, problems are resolved, and timely financial reporting is provided to each Sponsor. The OGA provides a significant number of services to KFRC scientists which fall under the broad categories of: Pre-Award (RFP/PA reviews, budget development, liaison with collaborating sites, proposal preparation, editing, and submission); Post-Award (A-110 and A-122 compliance, preparation and negotiation of subawards/subcontracts, award management, no-cost extensions, and closeouts); and Finance (fiscal oversight, A-133 compliance, a contact point for sponsors or Federal/State or other agencies on financial matters, and financial reporting to each sponsor). The OGA reviews and negotiates (in conjunction with the Finance Office and our legal counsel) all industry-sponsored, clinical research contracts and agreements, and it provides administrative oversight of entries and updates pertinent to our clinical research studies by utilizing ClinicalTrials.gov. Finally, the OGA works closely with the Institutional Review Board Office to ensure that all projects/studies that involve human subjects receive the requisite approvals.

Institutional Review Board (IRB)

The Institutional Review Board (IRB) is an official committee of Kessler Foundation Research Center, made up of scientists and nonscientists, whose function is to protect participants in research studies at Kessler Foundation Research Center (KFRC) and Kessler Institute for Rehabilitation (KIR). The IRB provides an independent review of all research studies involving patients and healthy volunteers at KFRC and KIR to assure 1) that potential risks to the research subjects are minimized and acceptable in relation to potential benefits and 2) that the subjects are given understandable information about the study so their decision to participate in the study is truly informed.

Before a research study can be initiated at KFRC and KIR, it must first be submitted to the IRB for its review and approval. Only IRB members who are not involved in the study are allowed to vote

on its approval. If the risks to participants are minimal in comparison to potential benefits and if the informed consent document is written in clear, non technical language, the study is approved. Many studies are conditionally approved pending changes requested by the IRB to clarify the language of the consent form so that potential research subjects can provide truly informed consent. Studies are approved for one year and must be re-evaluated by the IRB annually. Any changes to the study procedures must be reviewed and approved by the IRB before they are implemented. Any adverse events that occur to research subjects must be submitted to the IRB for its assessment of whether the event reflects a potential risk to other study participants.

The IRB is a crucial part of a system that protects participants in research studies. The leaders of KFRC and KIR have provided written assurances to the secretary of Health and Human Services that all human research carried out at these institutions will be performed with the highest ethical standards. The IRB's role is to implement these assurances.

Information Systems and Technology (IST)

KFRC research activities are secured and supported by a sophisticated network of computers and support peripherals linked through a common Ethernet network. The dedicated, secure Internet connection provides high-bandwidth connectivity for web sites, web-based services, and specialized research connectivity to affiliated university laboratories, and desktop video conferencing for "tele-rehabilitation" and virtual reality research. The local area network (LAN) includes over 120 workstations in the research laboratories, the Medical Library, the Conference Center, and the Education offices. Workstations in the SCI Research Laboratory are equipped with all Microsoft office applications, a number of statistical software packages, including STATA, PARSCALE, SPSS, HLM and Winsteps, reference management (EndNote), database management software (Access), qualitative analysis software (NVivo), stimulus delivery software (E-Prime), Dragon Naturally Speaking, and Adobe Acrobat. Tablet computers are also available to provide multi-modal usage options for persons with SCI. KFRC labs also benefit from central information technology equipment specification, procurement, installation, configuration, and on-demand technical support for advanced data management and analysis. The IT group at KF consists of five full-time staff. This group designed and manages KesslerFoundation.org that describes the full spectrum of our research in the field of rehabilitation.

Library Resources

A full-time medical librarian and a part-time library assistant staff the Kessler Medical Library and Patient Resource Center (MLPRC), located on the West Orange KIR campus. The MLPRC provides resources in print and electronic formats, and supports all research and education activities of KFRC and affiliated agencies. The library holdings emphasize specialized publications pertaining to the field of Physical Medicine and Rehabilitation. The collection includes approximately 1,500 texts and nearly 150 journal titles with more resources accessible electronically or in hard copy through inter-library loan. In addition, there is a consumer health collection designed for the general public and for patients and their families. Internet reference resources available include MEDLINE, Cochrane, OVID Access License, PSYCHINFO, and CINAHL. An extensive collection of electronic and print resources is also available to KFRC researchers through the Rutgers University library system, which houses the major health sciences research library for the State of New Jersey. Access to the Libraries Health Information Network is available to faculty and staff, either on-site or remotely.
